# Development of a miRNA-controlled dual-sensing system and its application for targeting miR-21 signaling in tumorigenesis

**DOI:** 10.1038/s12276-020-00537-z

**Published:** 2020-12-11

**Authors:** Yoona Seo, Sung Soo Kim, Namdoo Kim, Sungchan Cho, Jong Bae Park, Jong Heon Kim

**Affiliations:** 1 Department of Cancer Biomedical Science, Graduate School of Cancer Science and Policy, National Cancer Center, Goyang, 10408 Republic of Korea; 2 Division of Cancer Biology, Research Institute, National Cancer Center, Goyang, 10408 Republic of Korea; 3 Division of Clinical Research, Research Institute, National Cancer Center, Goyang, 10408 Republic of Korea; 4 Voronoi Bio Inc., Incheon, 21984 Republic of Korea; 5 Targeted Medicine Research Center, Korea Research Institute of Bioscience and Biotechnology, Ochang, 28116 Republic of Korea

**Keywords:** Oncogenes, Cell signalling

## Abstract

MicroRNAs (miRNAs) are considered to be strong prognostic markers and key therapeutic targets in human diseases, especially cancer. A sensitive monitoring platform for cancer-associated miRNA (oncomiR) action is needed for mechanistic studies, preclinical evaluation, and inhibitor screening. In this study, we developed and systemically applied a sensitive and efficient lentivirus-based system for monitoring oncomiR actions, essentially miR-21. The specificity and sensitivity of “miRDREL” against various oncomiRs were validated by checking for tight correlations between their expression and targeting efficacy. Experiments based on the transfection of synthetic mimics and antagomir-mediated depletion of oncomiRs further confirmed the specificity of the system. Systemic application of miRDRELs to natural oncomiR targets, knockdown of key microprocessors, and physiological triggering of oncomiRs also demonstrated that the system is an effective tool for monitoring cellular oncomiR action. Importantly, molecular modeling-based screening confirmed the action of the miR-21-targeting drug ivermectin and led to the identification of a new effective derivative, GW4064, for inhibiting oncogenic DDX23-miR-21 signaling. Furthermore, proteomic-kinase inhibitor screenings identified a novel oncogenic kinome-DDX23-miR-21 axis and thus expands our understanding of miR-21 targeting therapeutics in tumorigenesis. Taken together, these data indicate that miRDREL and its versatile application have great potential in basic, preclinical studies and drug development pipelines for miRNA-related diseases, especially cancer.

## Cancer research: Tracking microRNA molecules

A new method for monitoring microRNAs (miRNAs), very short RNA molecules that regulate gene expression, shows promise for developing and testing new cancer therapies. These miRNAs are strongly implicated in cancer, and are used for diagnosis and as therapeutic targets. However, currently available systems for monitoring them are inefficient and lack capacity for scaling up. Jong Heon Kim and co-workers at the National Cancer Center in Goyang, South Korea, have developed a new miRNA monitoring method that can be used in multiple disease models, including long-term experiments in small animals. They used the method to clarify how the cancer drug ivermectin acts, to identify a molecule similar to ivermectin but that may be more effective, and to identify novel molecules that interact with cancer-related miRNAs. This method shows promise for both clinical and basic research applications.

## Introduction

MicroRNAs (miRNAs) are considered to be strong prognostic markers and key therapeutic targets in various human diseases, especially cancer^1^. More than 600 different miRNAs encoded in the human genome negatively regulate gene expression at the posttranscriptional level by inducing the translational repression and/or destabilization of specific mRNAs by targeting their 3′ untranslated region (UTR)^2,3^. A miRNA typically regulates gene expression by facilitating the binding of the Argonaute-2 (AGO2) protein to target sequences in transcripts. When the targeting site in mRNA is completely complementary to miRNA, AGO2 slices the target transcript^4,5^. Thousands of different genes are subject to regulation by a single miRNA or miRNA family^6^. Although the mechanisms of action and the oncogenic role of miRNAs are relatively well-understood in cancer, their monitoring tools, and therapeutic approaches are still under investigation.

The abilities to monitor and visualize the actions of miRNAs in various in vitro cellular contexts and in vivo animal systems are critical for elucidating the actions and functions of cancer-associated miRNAs (oncomiRs)^7,8^. Furthermore, the development of sensitive and efficient biosensing systems is in strong demand for diagnosis, systemic inhibitor screening, and drug design platforms for oncomiR-related cancer therapeutics^9,10^. Various conventional approaches for measuring miRNA biogenesis and action are currently available; however, some of the widely accepted methods for detecting and monitoring miRNAs, such as northern blotting and real-time reverse transcription-PCR (qRT-PCR), are not suitable for the high-throughput platforms needed for the screening of antitumorigenic pharmacological drugs^11–13^. Moreover, the currently available systems that transiently express vector/retrovirus-based sensing miRNA action suffer from various technical challenges, such as low transfection/infection efficiency, cell-type limitations, and low working efficacy^14–16^.

In this report, we describe a multipurpose lentivirus-based monitoring system that can be used for the assessment of oncomiR targeting, biogenesis, oncogenic action, preclinical studies, and systematic mechanism-based screening of antitumorigenic inhibitors. Our “miRDREL” (miRNA-controlled dual reporter-expressing lentivirus) system showed excellent performance and versatile application potential, especially in the context of oncomiR-related cancer models. This dual luminescence/fluorescence-based reporter system enables the monitoring of oncomiR action in a highly sensitive and specific manner and also offers researchers to easily normalize the activities relative to those of counter-directed promoter-driven reporters. Switchable cassettes can be optimized and used to overcome various hurdles for effectively monitoring oncomiR actions in an in vitro cellular context and in vivo small animal models. Furthermore, the operational specificity and sensitivity of this superb system were rigorously validated in various ways. Mechanism-based molecular modeling and proteomic-kinase inhibitor screenings of the most essential oncogenic miR-21 signaling pathway using this versatile system to identify a novel cellular antitumorigenic axis are discussed.

## Materials and methods

### Cell culture

The human embryonic kidney cell line 293T and the U87MG human glioma cell line were obtained from the American Type Culture Collection (Manassas, VA, USA). 293FT and Lenti-X 293T cells were purchased from Thermo Fisher Scientific (Waltham, MA, USA) and Takara Bio Inc. (Shiga, Japan), respectively. The A549 human non-small cell lung cancer cell line, PANC-1 human pancreatic cancer cell line, NCI-H1299 human metastatic lung cancer cell line, Huh7 human hepatocellular carcinoma cell line, and CSC2 human glioma stem cell line were obtained from various researchers as described previously^17–21^. The 293T, 293FT, Lenti-X 293T, U87MG, PANC-1, and Huh7 cells were cultured in Dulbecco’s modified Eagle medium (DMEM; HyClone - GE Healthcare, Chicago, IL, USA). The A549 and NCI-H1299 cells were cultured in RPMI 1640 medium (HyClone). The CSC2 glioma stem cells were cultured as described previously^20^. All media except that used to culture CSC2 were supplemented with 10% fetal bovine serum (HyClone), 1% penicillin/streptomycin (Welgene, Gyeongsan, Republic of Korea), and 10 μg/ml ciprofloxacin (Santa Cruz Biotech, Santa Cruz, CA, USA).

### Small RNA analysis by northern blotting

Total RNA was isolated with TRIzol (Ambion - Thermo Fisher Scientific), and northern blotting was carried out with *N*-(3-dimethylaminopropyl)-*N*′-ethylcarbodiimide hydrochloride (EDC; Sigma-Aldrich, St. Louis, MO, USA) cross-linking method. In brief, 2–10 μg of total RNA was separated by denaturing urea-12% PAGE and transferred onto Hybond-NX Nylon hybridization membranes (GE Healthcare, Chicago, IL, USA). The membrane was hybridized overnight at 42 °C with 5′-^32^P-end-labeled oligonucleotide DNA probes of anti-miR-21 (5ʹ-TCAACATCAGTCTGATAAGCTA-3ʹ), anti-let-7a (5ʹ-AACTATACAACCTACTACCTCA-3ʹ), anti-miR-7 (5ʹ-ACAACAAAATCACTAGTCTTCCA-3ʹ), anti-miR-122 (5′-CAAACACCATTGTCACACTCCA-3′), and anti-U6 (5′-GCTTCACGAATTTGCGTGTCATCCT-3′) in PerfectHyb Plus Hybridization Buffer (Sigma-Aldrich) and then washed according to standard procedures. Radioactive signals were scanned by a BAS-2500 (Fujifilm, Tokyo, Japan) analyzer or Typhoon FLA 7000 biomolecular imager (GE Healthcare) or obtained on X-ray film (AGFA, Mortsel, Belgium) exposed at −80 °C.

### Luciferase assay of miRDRELs

Various luminescence-based miRDREL-expressing cells were prepared by lentiviral infection and then harvested under specific experimental conditions, including posttransfection of RNA oligomers (siRNAs, miRNA mimics, and anti-miRNAs) and drug treatment. Cells were lysed with Passive Lysis Buffer (Promega, Madison, WI, USA), and an aliquot of each lysate was analyzed by measuring luminescence signals with a Dual-Luciferase Reporter Assay System (Promega) in a reader (SpectraMax L, Molecular Devices, Sunnyvale, CA, USA). The miRNA sensor signal from firefly luciferase was normalized by that from *Renilla* luciferase. The normalized quantification data were used as the relative luciferase activities.

### Mouse orthotopic and subcutaneous xenograft models

For the orthotopic mouse model, 1 × 10^4^ fluo-miRDREL-expressing U87MG cells were first resuspended in Dulbecco’s phosphate-buffered saline (DPBS) and then transplanted into the left striatum of 6-week-old female BALB/c nude mice by stereotactic injection. The injection coordinates were 2.2 mm to the left of the midline and 0.2 mm posterior to the bregma at a depth of 3.5 mm. The tumors were extracted, pooled for each experimental group, and mechanically disaggregated using stainless steel operating scissors. The brain of each mouse was harvested and fixed in 4% paraformaldehyde in phosphate-buffered saline. A subcutaneously transplanted xenograft model was established by injecting 1 × 10^5^ control and luc-21-miRDREL-expressing A549 cells subcutaneously into the hip area on both sides of nude mice. After 4 weeks, luminescence images were acquired by intratumoral injection with *Renilla* (ViviRen In Vivo *Renilla* Luciferase Substrate, Promega) and firefly substrates (D-luciferin sodium salt, Sigma-Aldrich). The subcutaneous xenograft mice were administered control vehicle or ivermectin (IVOMEC, Merial, Lyon, France) through an intratumoral route. The dose of ivermectin was 3 mg/kg. The imaging data were expressed by Living Image Software (IVIS Spectrum In Vivo Imaging System, PerkinElmer, Waltham, MA, USA) and analyzed by GraphPad Prism 7 (GraphPad Software, San Diego, CA, USA). All animal experiments were conducted in accordance with protocols approved by the Institutional Animal Care and Use Committee of the National Cancer Center, Republic of Korea. NCCRI is an Association for Assessment and Accreditation of Laboratory Animal Care International (AAALAC International)-accredited facility and abides by the Institute of Laboratory Animal Resources (ILAR) guide.

### Proliferation assay

Cells were plated at 1 × 10^4^ cells/well density in 96-well plates for in vitro proliferation assays. The luminescence of viable cells was detected using a CellTiter-Glo Luminescent Cell Viability Assay kit (Promega) according to the manufacturer’s protocol. The luminescence signal was detected by a SpectraMax L Microplate Reader (Molecular Device) according to the manufacturer’s protocol.

### Kinase inhibitor screening

A kinase inhibitor library, collection of 193 kinase inhibitors, and identified specific kinase inhibitors (HMN-214, AT7519, SNS-032, and KX2-391) were purchased from Selleckchem (Houston, TX, USA). All chemicals were dissolved in dimethyl sulfoxide (DMSO) before treatment, and the cells were treated with various concentrations. To screen small-molecule compounds that modulate luc-21-miRDREL A549 cells (1 × 10^4^ cells/well) in a 96-well plate were treated with 0.1, 0.2, 1, and 4 μM of kinase inhibitor library for 48 h. Cells were assayed with a Dual-Luciferase Reporter Assay System (Promega).

### Statistical analysis of data

Data are presented as the mean ± standard deviation determined from at least three independent experiments. Differences were assessed by two-tailed Student’s *t*-test using Excel software (Microsoft, Redmond, WA). *P* < 0.05 was considered statistically significant.

## Results

### Development of miRDRELs and their miRNA-targeting ability in cells

**Fig. 1:**
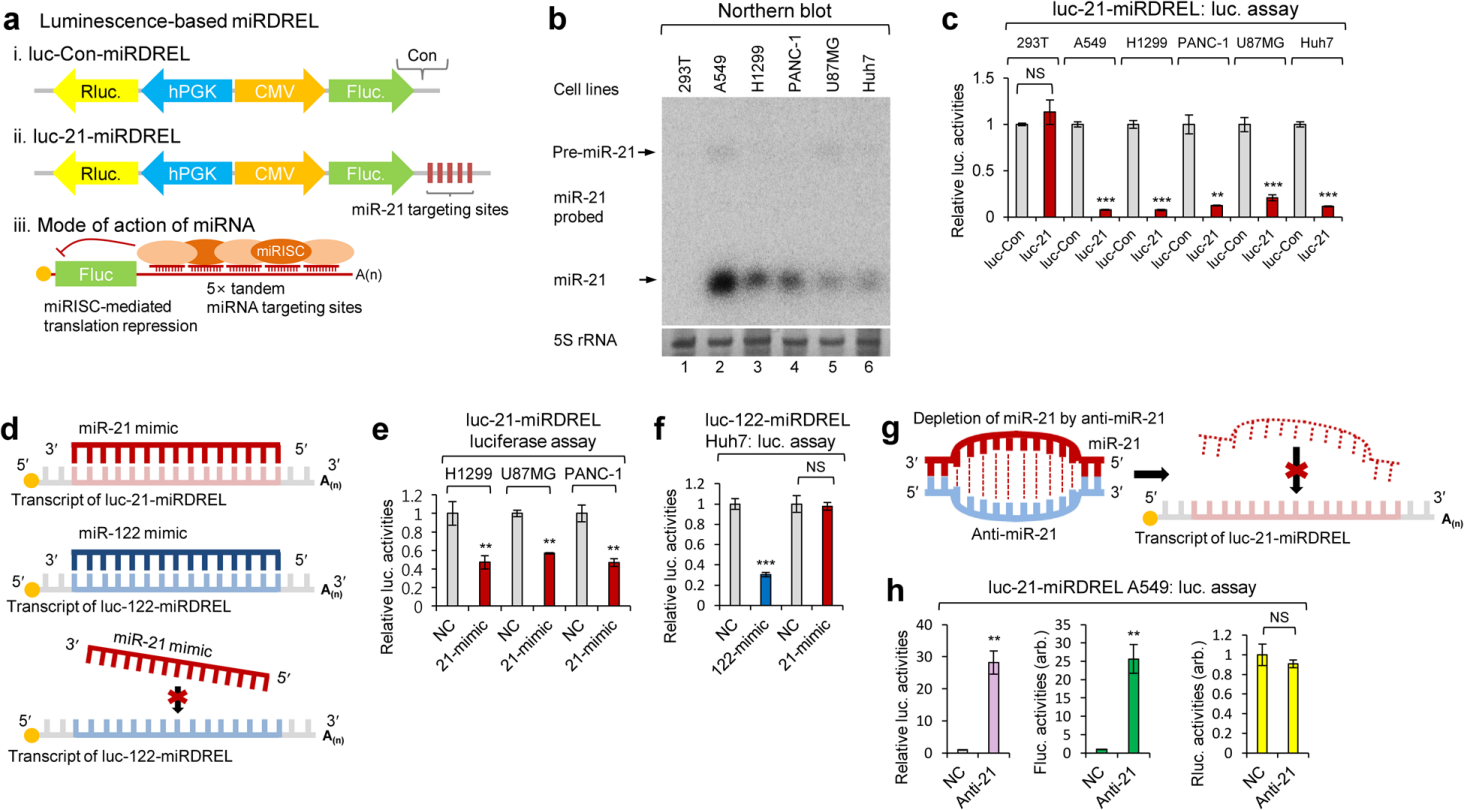
miRNA-targeting ability of the miRDREL system in cells. **a** Schematic depiction of luc-Con-miRDREL (i), luc-21-miRDREL (ii), and the mode of action of miRNA-targeting to the miRDRELs (iii). miRISC; miRNA-induced silencing complex. **b** Northern blot analysis of endogenous miR-21 in various cell lines, including human embryonic kidney (293T), non-small cell lung cancer (A549), metastatic lung cancer (NCI-H1299), pancreatic ductal adenocarcinoma (PANC-1), glioblastoma multiforme (U87MG), and hepatocellular carcinoma (Huh7) cells. The arrow indicates bands of precursor (pre-miR-21) and mature miR-21. 5S rRNA was used as a loading control. **c** Firefly luciferase activities showed an inverse correlation with the expression level of endogenous miR-21, which binds to the miRNA-targeting sites in the 3′UTR of firefly luciferase in each luc-21-miRDREL construct. Firefly luciferase activity was normalized by that of *Renilla* luciferase. The data represent the mean values of three independent experiments (*n* = 3). Error bars in the graph represent ±standard deviation, and the *P*-values are based on a comparison of luc-Con-miRDREL to luc-21- miRDREL. **P < 0.01, ***P < 0.001; NS, not significant. **d**–**h** Analysis of miRDREL specificity with synthetic miRNA mimics and anti-miRNAs. **d** Targeting and nontargeting principles of miRNA mimics (miR-21 and miR-122-mimics) to miRDRELs and the schematic depiction of mimicry for each miRNA (miR-21 and miR-122). **e**, **f** Inhibition of firefly luciferase activities indicates that each miRNA mimic binds to the miRNA-targeting sites in the luc-miRDREL constructs. In contrast, unaltered luciferase activities indicate that the miRNA mimic does not bind to miRDREL and it is noncompatible to miRNA-targeting sites. The data represent the mean values of three independent experiments (*n* = 3). Error bars in the graph represent ±standard deviation, and the *P*-values are based on a comparison of the negative control (NC) to each mimic (miR-21 and miR-122 mimics). **P < 0.01, ***P < 0.001; NS, not significant. **g** Depletion strategy of endogenous miR-21 with anti-miR-21 in cells. **h** luc-21-miRDREL-expressing A549 cells were transfected with synthetic anti-miR-21 (anti-21) or its relevant negative control oligomer (NC). Specific depletion of endogenous miR-21 via synthetic anti-miR-21 transfection specifically induced the derepression of firefly luciferase activities but not that of the normalization control, *Renilla* luciferase. The data represent the mean values of three independent experiments (*n* = 3). Error bars in the graph represent ±standard deviation, and the *P*-values are based on a comparison of the NC to anti-miR-21. **P < 0.01; NS, not significant; arb., arbitrary unit.

As shown in Fig. 1a, the miRDREL system operates on a miRNA-targeting principle. Generally, miRNA targeting occurs through its association with the 3′UTR of a target mRNA to cause translational silencing and/or deadenylation^2,3^. Conceptually, high expression of a specific miRNA induces strong translational repression of a reporter gene; hence, high miRNA expression is used to induce an “off” signal. In contrast, low or null expression of a specific miRNA induces derepression of the reporter to induce an “on” signal. Therefore, the altered translational regulation of a miRNA target linked to reporter activity can be easily monitored in vitro and in vivo by measuring the strength of luminescence or fluorescence (Supplementary Fig. 1).

After generating luc-miRDRELs (Supplementary Table 1), we assessed their ability to target representative oncogenic miR-21 and other essential oncomiRs (let-7a, miR-7, and miR-122)^22–27^. First, we used northern blotting to examine the expression levels of these oncomiRs in various cancer cell lines [non-small cell lung cancer (A549 cells), metastatic lung cancer (NCI-H1299 cells), pancreatic cancer (PANC-1 cells), glioblastoma multiforme (U87MG cells), hepatocellular carcinoma (Huh7 cells)] and the SV40 large T antigen-transformed human embryonic kidney cell line (293T cells) (Fig. 1b and Supplementary Fig. 3).

As shown in Fig. 1b and Supplementary Fig. 3b, miR-21 and let-7a were maximally expressed in A549 cells and negligibly detectable in 293T cells. The translational silencing ability of endogenous miR-21 and let-7a was tested by infection of the designated cell lines with miRNA-targeting site null (luc-Con-miRDREL), miR-21-targeting site bearing (luc-21-miRDREL), and let-7a-targeting site bearing (luc-let-7a-miRDREL) miRDRELs. The translational repression of luc-21-miRDREL and luc-let-7a-miRDREL showed relative linearity similar to the expression levels of endogenous miRNAs in the various cancer cell lines tested. Conversely, neither luc-21-miRDREL nor luc-let-7a-miRDREL exhibited repression in 293T cells (Fig. 1c and Supplementary Fig. 3c). luc-let-7a-miRDREL showed a slight repression effect in Huh7 cells; although our northern blot analysis failed to detect let-7a in Huh7 cell total RNA, we detected let-7a expression in this cell line using small RNA enrichment (data not shown)^28^. Taken together, these data indicate that our miRDREL system operates well and exhibits specificity for each of the tested oncomiRs in cancer cell lines.

### Analysis of miRDREL specificity with synthetic miRNA mimics and anti-miRNAs

Next, the specificity of each luc-miRDREL against synthesized miRNAs was determined (Fig. 1d) by testing the transfection of the appropriate RNA oligomers into luc-miRDREL-bearing 293T, A549, Huh7, NCI-H1299, U87MG, and PANC-1 cells. As shown in Fig. 1e, the synthetic miR-21 mimic specifically and severely inhibited the translation of firefly luciferase in luc-21-miRDREL-bearing NCI-H1299, U87MG, and PANC-1 cells, but not in the cells transfected with the negative control (NC) or a null (irrelevant sequence) version of luc-21-miRDREL.

To exclude potential off-target effects, we introduced synthetic miR-21 mimics as miRNA mimic controls against luc-122-miRDREL. As shown in Fig. 1f, luc-122-miRDREL-expressing Huh7 cells did not respond to irrelevant mimics (NC and miR-21 mimics), which targeted irrelevant “seed” sequences^29^. These results clearly demonstrated that each luc-miRDREL shows specificity solely for its target miRNA in cells. Using similar strategies, we also essentially verified the specificities of luc-7-miRDREL and luc-21-miRDREL in various cell lines (293T, A549, Huh7, U87MG, and PANC-1 cells). As shown in Supplementary Fig. 4a-f, each miRDREL showed specificity for its intended target and did not respond to irrelevant miRNA.

We next sought to deplete endogenous miRNAs using synthetic anti-miRNA oligomers (antagomirs) that had specific chemical modifications and perfect sequence complementarity to corresponding endogenous miRNAs (Fig. 1g). As shown in Fig. 1b, miR-21 exhibited the highest expression level in A549 cells and was moderately expressed in PANC-1 cells. As expected, depletion of endogenous miR-21 from these cell lines yielded significant restoration of the luciferase activities derived from luc-21-miRDREL. Firefly luciferase activity was derepressed by approximately 25-fold in the A549 cells (Fig. 1h) and ~10-fold in the PANC-1 cells (Supplementary Fig. 4h), suggesting that the anti-miR-21 completely depleted or blocked the reporter targeting action of miR-21 in these cells. There was no change in *Renilla* luciferase activity in either case, demonstrating that anti-miR-21 specifically targeted endogenously expressed miR-21 and specifically depleted miR-21 action in these cells (Fig. 1h and Supplementary Fig. 4 h). Together, these data indicate that our luc-miRDREL system operates well and exhibits great specificity for each of the tested miRNAs in various cellular contexts of cancer.

### Analysis of cellular oncomiR-targeting site bearing miRDRELs

A “seed” sequence is a heptameric sequence that is conserved among species and most often situated at nucleotide (nt) positions 2~7 from the 5′-end of miRNA; it is essential that a miRNA bind to its mRNA target^29^. Therefore, in addition to testing with perfectly matched sequences, the efficacy of our system with natural miRNA targets needed to be tested to ensure it overcomes potential problems due to species barriers, tissue specificity, etc. To perform these tests, we analyzed miRDRELs with natural miRNA-targeting sites derived from conventional mRNA 3′UTRs instead of synthetic, perfect complementary matches. Representative miRNA-targeting site bearing genes were selected based on the following criteria: (1) the presence of at least two miRNA-targeting sites for strong reporter inhibition^30–32^; (2) miRNA-targeting sites locate within 100-nt of one another to enable the efficient formation of a miRNA-induced silencing complex (miRISC)^33^; and (3) mRNA targeting was experimentally validated with the selected miRNAs. We screened the TargetScan7.2 miRNA-targeting database and selected mRNAs that satisfied these criteria.

**Fig. 2:**
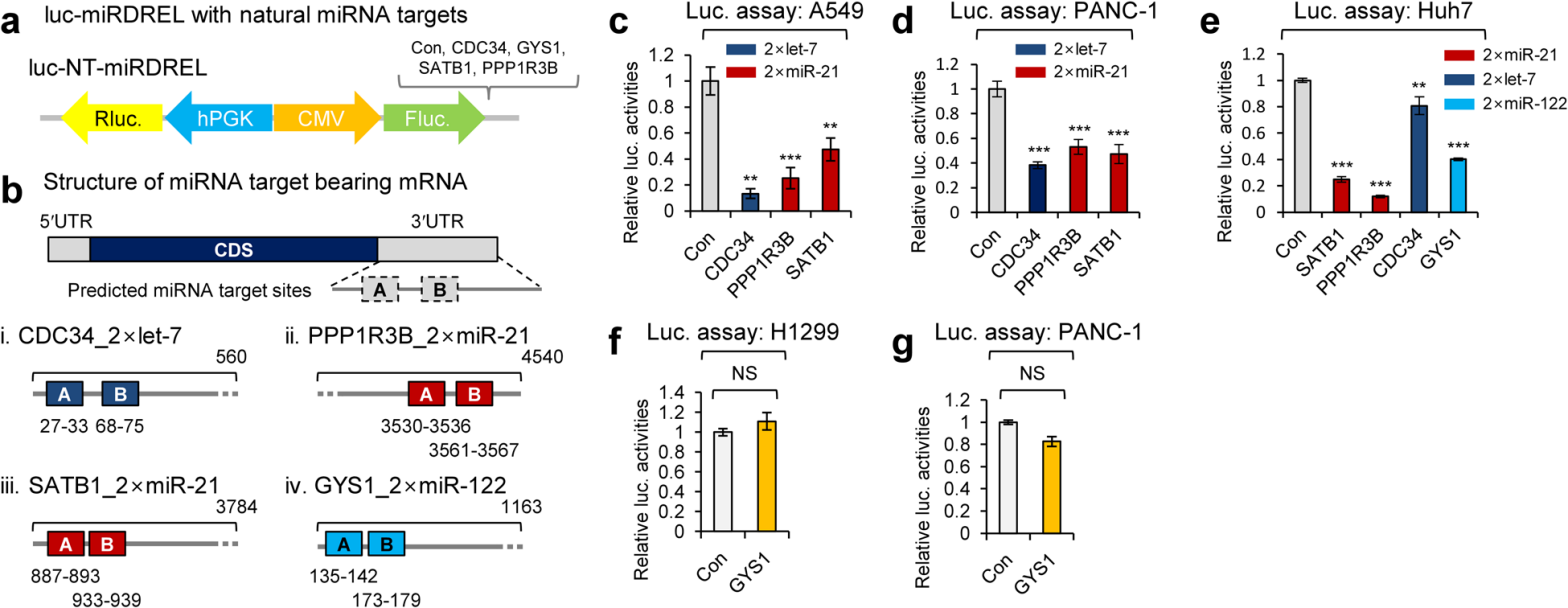
Analysis of cellular oncomiR-targeting site bearing miRDRELs. **a** Two consecutive cellular miRNA-targeting sites of let-7, miR-21, and miR-122 in ubiquitin-conjugating enzyme E2 R1 (CDC34), protein phosphatase 1 regulatory subunit 3B (PPP1R3B), SATB homeobox 1 (SATB1), and glycogen synthase 1 (GYS1) mRNAs were inserted into the firefly 3′UTR of luc-miRDREL. The relative distance and location of each miRNA-targeting site within the 3′UTR are depicted in panel (**b**). NT, natural miRNA targets. **c**–**e** Luciferase activities of each luc-miRDREL were compared to that of the target site null control. Repression of luciferase activity indicated that each luc-miRDREL was targeted by endogenously expressed let-7a, miR-21, or miR-122 in various carcinomas. The data represent the mean values of four independent experiments (*n* = 4). Error bars in the graph represent ±standard deviation, and the *P*-values are based on a comparison of the control to luc-NT-miRDRELs. **P < 0.01, ***P < 0.001. **f**, **g** Analysis of the cellular miR-122-targeting site bearing luc-GYS1-miRDREL in cells not expressing miR-122. The luciferase activity of luc-GYS1-miRDREL was not changed in the NCI-H1299 or PANC-1 cells, which do not express miR-122. The luciferase activity of luc-GYS1-miRDREL was compared to that of the miRNA-targeting site null control. The data represent the mean values of three independent experiments (*n* = 3). Error bars in the graph represent ±standard deviation. NS, not significant.

Cell division cycle 34 (CDC34; 2× let-7-targeting sites)^34^, protein phosphatase 1 regulatory subunit 3B (PPP1RB; 2× miR-21-targeting sites), special AT-rich sequence-binding protein 1 (SATB1; 2× miR-21-targeting sites)^35^, and glycogen synthase 1 (GYS1; 2× miR-122-targeting sites) were chosen for the analysis (Fig. 2a, b) (Supplementary Table 1). The generated miRDRELs were transduced into various cancer cell lines, and their specificities and activities were extensively measured. In the tested cell lines, all of the generated miRDRELs showed repressive tendencies similar to those observed for the systems bearing perfectly matched targeting sites (Fig. 2c–e). The natural miRDRELs did not show translational repression of the firefly luciferase reporter when transduced into cells that did not express the target miRNA (e.g., miR-122; Fig. 2f, g). Taken together, these results clearly demonstrate that the miRDREL system is also effective for monitoring the behavior of natural miRNA targets in general cancer cell lines.

### Analyzing the specificity and controllability of miRDRELs via the depletion of microprocessors and physiological triggering of oncomiR expression

**Fig. 3:**
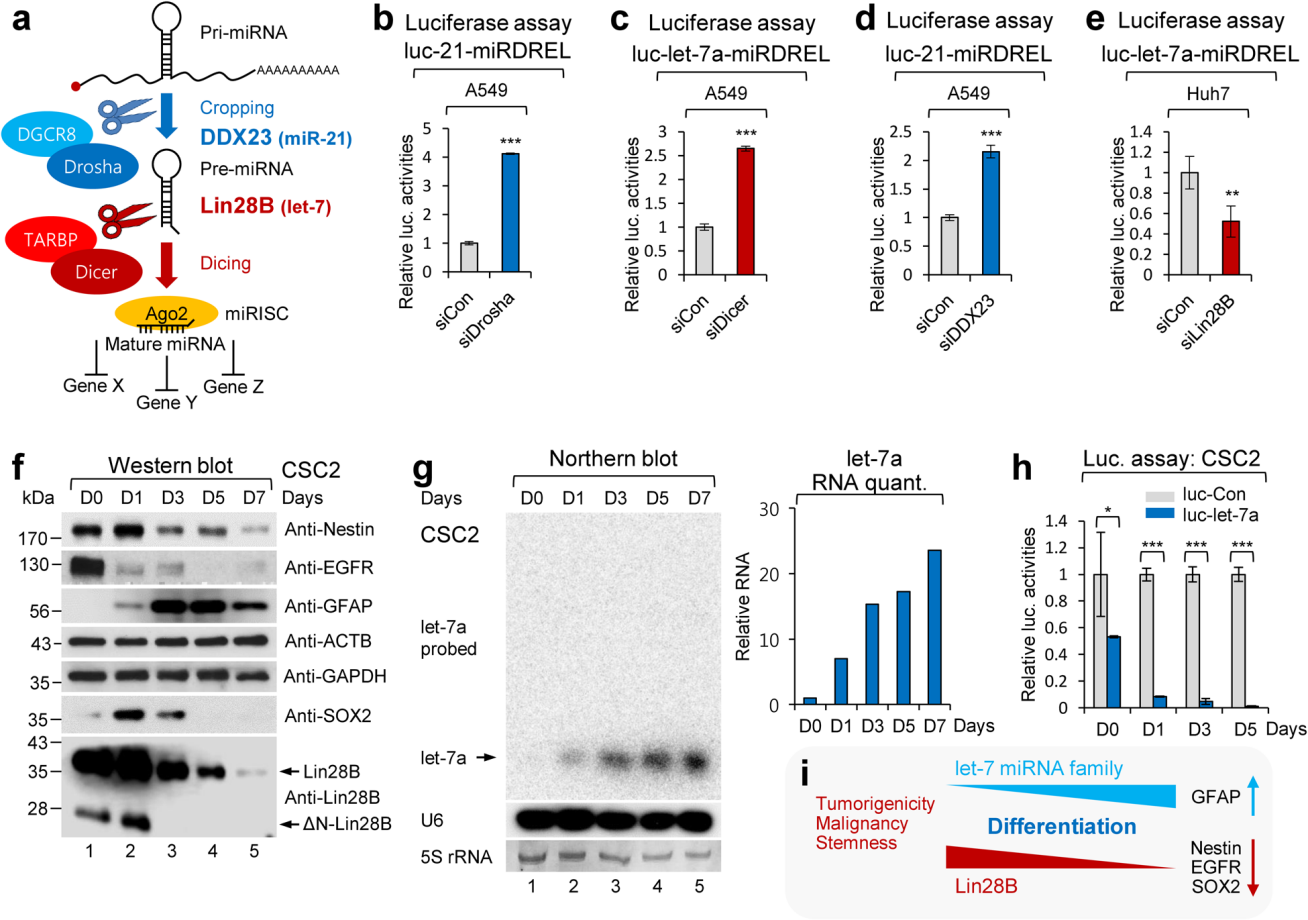
Analyzing the specificity and controllability of miRDREL via the depletion of microprocessors and physiological triggering of oncomiR expression. **a** Schematic diagram of the general miRNA-processing pathway by core miRNA processors (Drosha/DGCR8 and Dicer/TARBP) and auxiliary factors (DDX23 and Lin28B). Depletion of core microprocessors Drosha and Dicer derepressed the firefly luciferase activity of luc-21-miRDREL and luc-let-7a-miRDREL in A549 cells, respectively. **b**–**e** Luciferase assay results of each siRNA transfected miRDREL. **b** siRNAs against human Drosha (siDrosha) and control siRNA (siCon) were transfected into luc-21-miRDREL-expressing A549 cells. **c** siRNAs against human Dicer (siDicer) and control siRNA (siCon) were transfected into luc-let-7a-miRDREL-expressing A549 cells. **d** siRNAs against human DDX23 (siDDX23) and control siRNA (siCon) were transfected into luc-21-miRDREL-expressing A549 cells. **e** siRNAs against human Lin28B (siLin28B) and control siRNA (siCon) were transfected into luc-let-7a-miRDREL-expressing Huh7 cells. The data represent the mean values of four independent experiments (*n* = 4). Error bars in the graph represent ±standard deviation, and the *P*-values are based on a comparison of the control to the siRNA of each microprocessor. **P < 0.01, ***P < 0.001. **f**–**i** Analysis of luc-let-7a-miRDREL behavior during differentiation induction in CSC2 glioma stem cells (GSCs). CSC2 cells were induced to differentiate by serum factors for 7 days. **f** Western blot analysis was assessed to identify the differentiation status of CSC2 cells using anti-Nestin, anti-EGFR, anti-GFAP, anti-SOX2, and anti-Lin28B antibodies. ACTB and GAPDH were used as internal loading controls. ΔN-Lin28B, N-terminal isoform of Lin28B. **g** Northern blot analysis and RNA quantification of endogenous let-7a expression during a serial differentiation period. The position of the arrow indicates mature let-7a miRNA. 5S rRNA and U6 were used as loading and hybridization controls, respectively. **h** Luciferase assay results of luc-let-7a-miRDREL-expressing CSC2 cells during differentiation. A luciferase assay with differentiated CSC2 cells was carried out at serial time points. The data represent the mean values of three independent experiments (*n* = 3). Error bars in the graph represent ±standard deviation, and the *P*-values are based on a comparison of luc-Con-miRDREL to luc-let-7a-miRDREL. *P < 0.05, ***P < 0.001. **i** Schematic diagram for the relationship between the let-7 miRNA family and differentiation-related factors in GSCs.

Typical miRNAs are ~22-nt in length. Mature miRNA is generated from a longer precursor called a precursor miRNA (pre-miR), which is originally processed from a primary miRNA (pri-miR) transcript. Most pri-miR transcripts are transcribed by RNA polymerase II and then capped and polyadenylated. Unlike general mRNAs, pri-miRs are cleaved in the nucleus by a Drosha/DGCR8 microprocessor that recognizes a specific stem-loop structure. Pre-miRs are transported out of the nucleus and subsequently processed to mature miRNAs by the cytoplasmic microprocessor Dicer/TARBP (Fig. 3a)^36^.

To check whether the miRDREL system properly responds to the core miRNA processors Drosha and Dicer, we depleted these molecules with small interfering RNAs (siRNAs) in luc-21-miRDREL- and luc-let-7a-miRDREL-expressing A549 cells. As shown in Fig. 3b, c, depletion of Drosha and Dicer led to marked induction of firefly luciferase activities, reflecting the disruption of endogenous miR-21 processing. These data indicate that the miRDREL system is specifically reactive to the general miRNA biogenesis pathway. Moreover, the specific depletion of DDX23, which is an positive oncogenic modulator of pri-miR-21 processing, showed a similar derepression pattern^19^ (Fig. 3d). In contrast, the specific depletion of Lin28B, which inhibits let-7 miRNA family member processing^37^, led to enhanced repression of luc-let-7a-miRDREL in Huh7 cells. This result was consistent with the idea that let-7a miRNA expression is elevated by the specific depletion of its negative processing modulator, Lin28B (Fig. 3e).

The relationship between let-7 miRNA family members and the negative processing modulator Lin28B has been relatively well studied^37^. Lin28B expression tends to decrease during differentiation and generally shows an inverse correlation with the level of the mature let-7 miRNA family (Fig. 3i). As shown in Fig. 3f, the expression of Lin28B in glioma stem cells (GSCs; CSC2 cells) gradually decreased upon the induction of differentiation. The differentiation-related GSC markers Nestin, EGFR, and SOX2 were also markedly decreased. In contrast, the glial cell marker GFAP showed enhanced expression as differentiation progressed^20^. The expression of let-7a miRNA increased linearly upon differentiation induction and thus was highly correlated with the downregulation of Lin28B (Fig. 3g). Considering these observations, we tested the specificity and controllability of luc-let-7a-miRDREL upon physiological triggering of let-7a miRNA induction in CSC2 cells. As shown in Fig. 3h, the activity of luc-let-7a-miRDREL in the CSC2 cells gradually decreased as differentiation progressed, and it was highly correlated with the downregulation of the Lin28B protein and the upregulation of mature let-7a miRNA. Taken together, these data indicate that luc-miRDREL exhibits specificity and controllability with respect to miRNA-processing machinery and the physiological triggering of miRNAs in patient-derived GSCs.

### Using miRDREL to visualize the actions of oncomiRs in vitro and in vivo

Recent studies have focused on using visualization techniques to detect and analyze cellular signaling noninvasively. In these cases, rather than performing quantitative biochemical end-point analyses, such as western blotting, qRT-PCR, northern blotting, or luminescence assays, it is preferable to detect the signal in a real or sequential timeline. Moreover, there are benefits to continuously observing signals without lysing cells or disrupting tissues^38^. Therefore, to visualize the behavior of miRNAs in cells, and even in tissues, of animal models, we developed a fluorescence sensor expressing miRDREL (fluo-miRDREL) by replacing the luminescence-based reporters with fluorescence reporters.

**Fig. 4:**
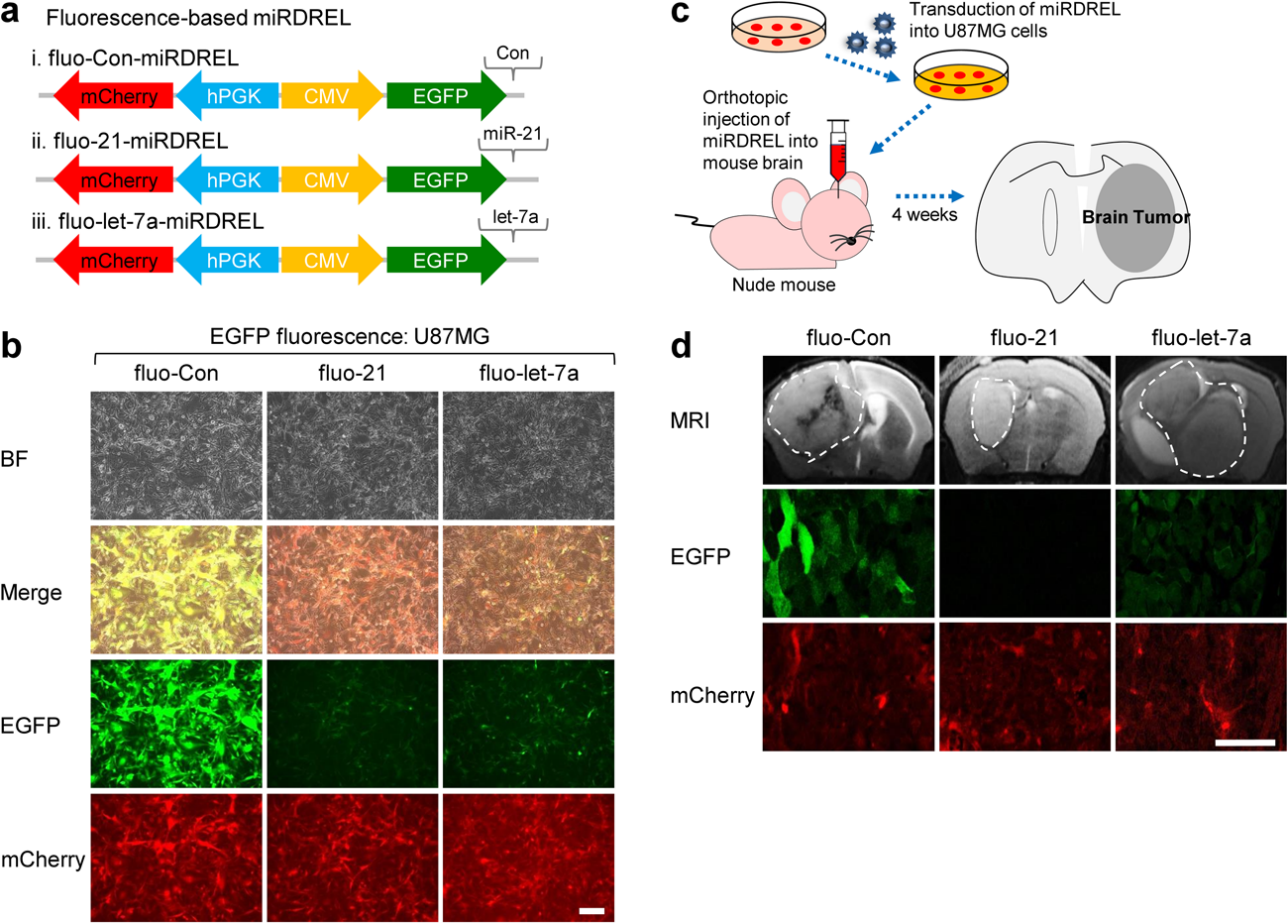
Using miRDREL to visualize the actions of oncomiRs in vitro and in vivo. **a** Schematic diagram of the control miRDREL (i, fluo-Con-miRDREL), fluo-21-miRDREL (ii), and fluo-let-7a-miRDREL (iii) for endogenous miR-21 and let-7 sensing and visualization. **b** Low EGFP signal from the systems (fluo-21-miRDREL and fluo-let-7a-miRDREL) represents high expression of endogenous miR-21 and let-7 in U87MG cells (scale bar: 200 µm). The experiments were repeated three times, and representative images are presented. BF, bright field image. **c** Establishment of an orthotopic mouse brain tumor model with fluo-miRDRELs for miRNA sensing and visualization. **d** Control-, miR-21-, and let-7a-fluo-miRDREL-expressing U87MG cells were injected into the mouse brain. After 4 weeks, the true formation of brain tumors was monitored by MRI, and then, the animals were sacrificed for the isolation of mouse tumor tissues. The mouse tumor tissues were prepared for immunohistochemistry, and then, the fluorescence signals were monitored to ensure fluo-miRDREL behavior. The EGFP signal in mouse brain tumor tissues stably expressing fluo-miRDRELs (fluo-21-miRDREL and fluo-let-7a-miRDREL) showed a reduction pattern similar to that of U87MG cells in vitro and confirmed the feasibility of the system for visualization in vivo (scale bar: 50 µm). mCherry expression was used as a control in vitro and in vivo. Areas marked with white dotted lines in the panels of the MRI scans represent tumors formed in the mouse brain. Four animals were used for each fluo-miRDREL experiment, and similar results were obtained. Representative images are presented in Fig. 4d.

As shown in Fig. 4a, firefly luciferase and *Renilla* luciferase were replaced with enhanced green fluorescence protein (EGFP) and mCherry, respectively. The fluo-miRDRELs were stably transduced into U87MG glioma cells, which showed relatively high and moderate expression levels of miR-21 and let-7a, respectively. As shown in Fig. 4b, the fluo-21- miRDREL and let-7a-miRDREL showed markedly reduced EGFP fluorescence intensity compared with the control sensor in vitro. The data showed similar patterns in the behavior of luc-miRDRELs for each miRNA.

We also sought to test the long-term working potential of a fluo-miRDREL and its relevant expression and monitoring of oncomiR behavior in an orthotopic mouse brain tumor model. fluo-miRDREL-expressing U87MG cells were injected into mouse brains, and after 4 weeks, brain tumor formation was monitored by magnetic resonance imaging (MRI). Mouse tumor tissues were isolated and prepared for immunohistochemistry, and the fluorescence signals were monitored (Fig. 4c). As shown in Fig. 4d, the expression of the fluorescence reporters in each miRDREL was sustained long-term in the animal tissues in vivo. Moreover, the in vivo results regarding the miRNA-targeting effect of two independent systems, fluo-21-miRDREL and let-7a-miRDRELs, were similar to those obtained in vitro. These data verify that our fluo-miRDRELs showed long-term efficacy for monitoring oncomiRs in an in vivo animal tumor model. Taken together, these data indicate that our system can be used to visualize oncomiR action efficiently in animal tissues as well as in cells.

### miRDREL-based in vivo imaging of the response to the miR-21 inhibitor ivermectin and identification of a new inhibitory derivative

Luminescence-based imaging of a living small animal using our new system is expected to open up new research avenues because it would avoid the need to sacrifice the animal for analysis. Sensitive detection of reporter activity in the living context is relatively well established^39–41^, and luminescence seems to be an appropriate method for visualizing and quantifying the effect of oncomiR inhibitors in vivo^38^.

Our group and others previously have shown that miR-21 expression is closely correlated with the malignancy of various cancers, and miR-21 is generally considered to act as an oncogene by negatively regulating various tumor-suppressive target mRNAs^19,22,23,42–44^. Thus, a strategy aimed at inhibiting the expression and/or biogenesis of miR-21 could contribute to managing tumor malignancy, making miR-21 an attractive target for drug development.

**Fig. 5:**
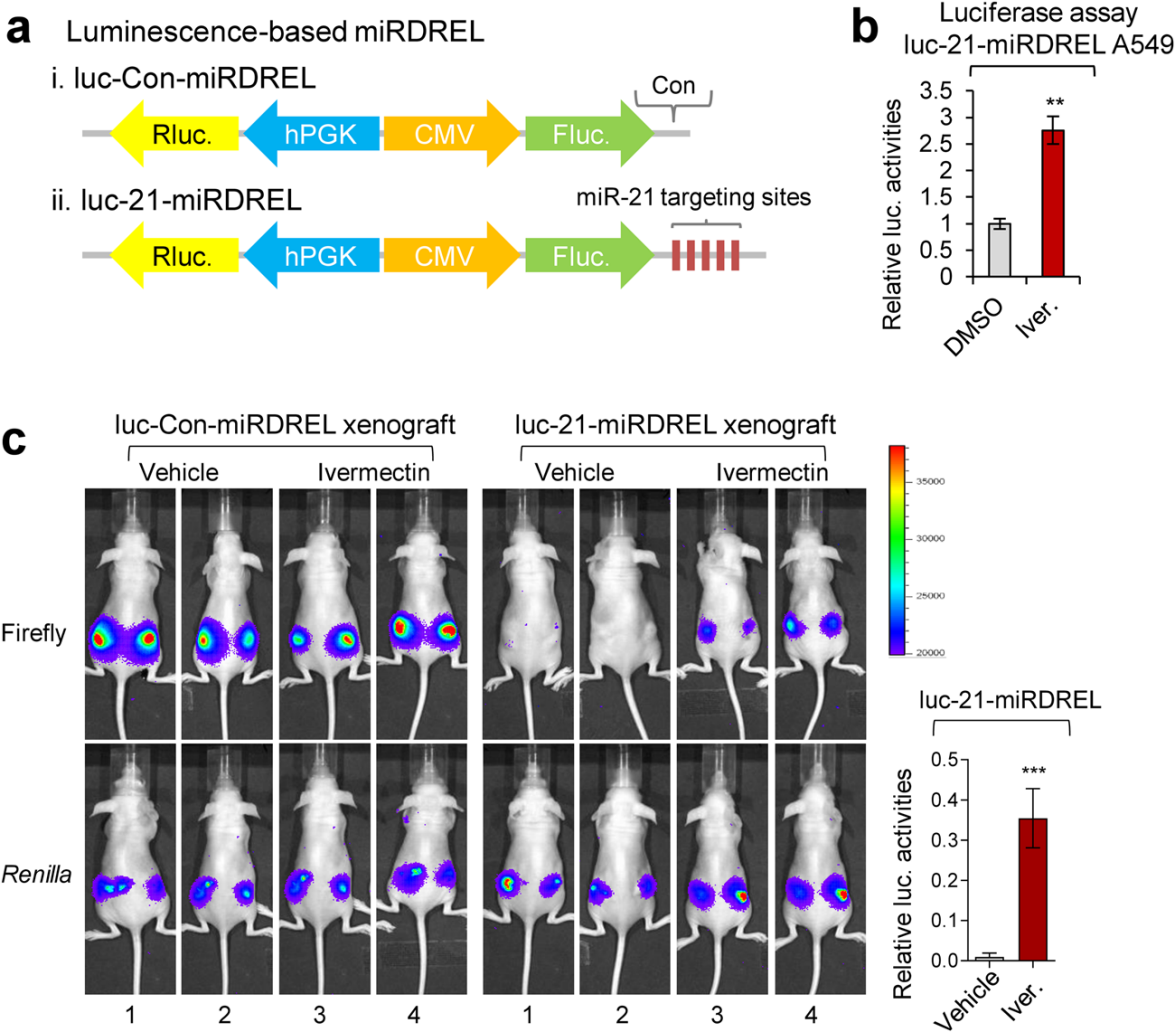
miRDREL-based in vivo imaging of the response to the miR-21 inhibitor ivermectin. **a** Schematic diagram of the luminescence-based control (i) and 21-miRDREL (ii) for small animal imaging and high-throughput screening. **b** Luciferase assay of luc-21-miRDREL-expressing A549 cells treated with ivermectin. Derepressed firefly luciferase activity shows the inhibition of miR-21 biogenesis by ivermectin in the luc-21-miRDREL-expressing A549 cells. The data represent the mean values of four independent experiments (*n* = 4). Error bars in the graph represent ±standard deviation, and the *P*-values are based on a comparison of the control (DMSO) to ivermectin. **P < 0.01. **c** In vivo imaging of ivermectin action on luc-miRDRELs in mice. Intratumoral administration of vehicle or ivermectin was assessed into the luc-miRDREL-expressing A549 tumors in xenograft mice. luc-Con-miRDREL A549 tumors showed constant firefly and *Renilla* luciferase activities in the subcutaneous mouse tumor model. In contrast, the luc-21-miRDREL A549 tumors showed nondetectable firefly luciferase activities upon vehicle treatment, which indicates the repression of the firefly luciferase sensor by endogenous miR-21 in tumors. Restoration of the firefly luciferase activity indicates that endogenous miR-21 biogenesis was perturbed by the ivermectin treatment. Error bars in the graph represent ±standard deviation. The *P*-values are based on a comparison of the vehicle and ivermectin treatment in the luc-21-miRDREL-expressing xenograft set. *n* = 6, ***P < 0.001 (=0.0009).

We previously showed that the anti-parasite drug ivermectin blocks pri-miR-21 processing by decreasing the activity of the cellular oncogenic RNA helicase, DDX23^19^. Here, we examined the operation of luc-21-miRDREL upon ivermectin treatment (Fig. 5a). As expected, luc-21-miRDREL-expressing A549 cells showed strong induction of luciferase activity following exposure to ivermectin (Fig. 5b). To check the exact effect of ivermectin on pri-miR-21 processing in vivo, we performed long-term tumor imaging with a mouse xenograft model^45^. To test whether our system is suitable for this purpose, luc-Con-miRDREL- and luc-21-miRDREL-expressing A549 cells were subcutaneously injected into the flanks of nude mice. As shown in Fig. 5c, Supplementary Fig. 5, and consistent with our in vitro observation that ivermectin abrogates miR-21 processing, ivermectin-treated tumors showed higher (derepressed) expression of firefly luciferase activity (see Fig. 5c, ivermectin in the luc-21-miRDREL xenografts). However, vehicle-treated tumors showed no firefly luciferase signal in tumor regions (see Fig. 5c, vehicle in the luc-21-miRDREL xenograft). This result demonstrates that our miRDREL reflected the action of the miR-21 inhibitor ivermectin under long-term tumorigenic circumstances in vivo.

**Fig. 6:**
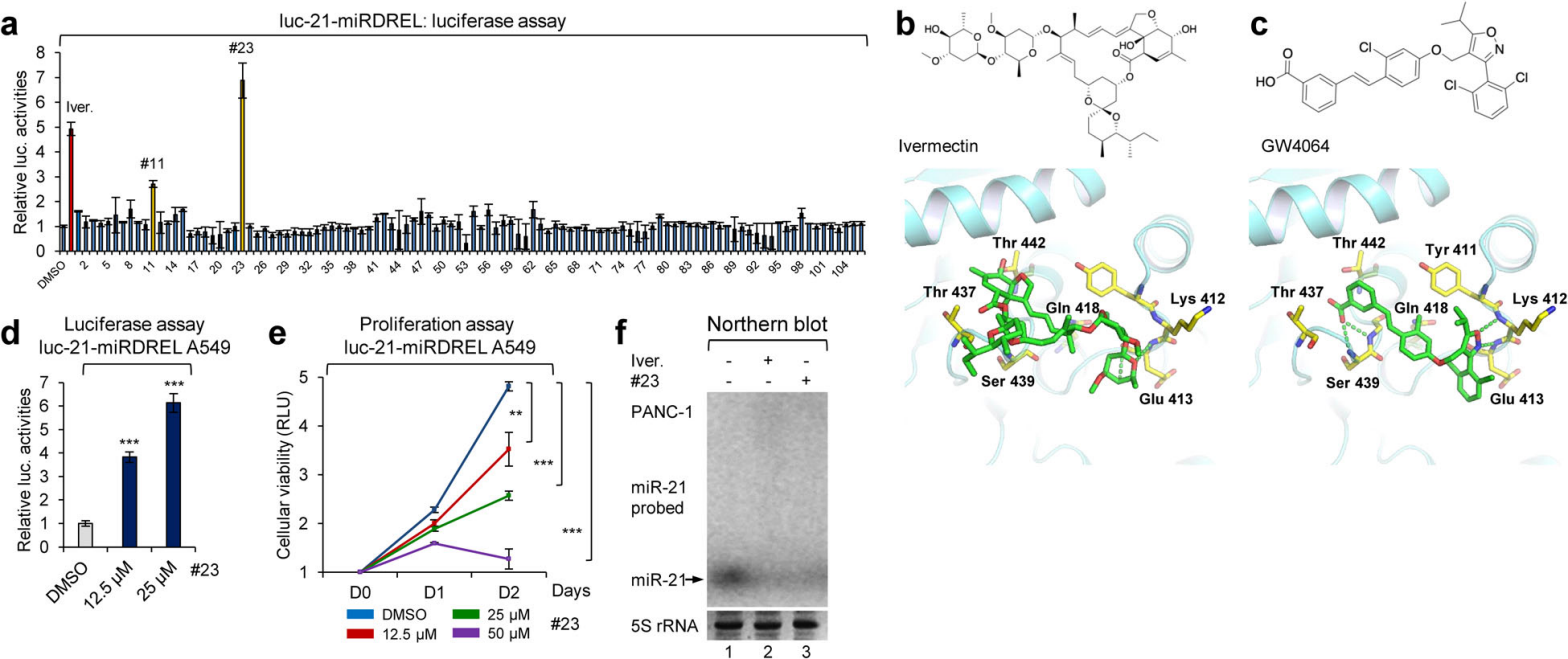
Molecular modeling and miRDREL-based identification of a new miR-21 inhibitory derivative, GW4064. **a** High-throughput screening of 106 ivermectin derivatives and the identification of compounds #11 and #23 (GW4064) as candidates for new miR-21 biogenesis inhibitors. **b** Chemical structure of ivermectin, which binds in the ATP-binding pocket of the pri-miR-21-processing effector DDX23. Molecular docking model of DDX23-ivermectin (bottom). Dashed lines represent hydrogen bond interactions between DDX23 and ivermectin. **c** Chemical structure of compound #23 (GW4064) based on molecular docking modeling-based library screening and the molecular docking model of DDX23-GW4064 (bottom). Dashed lines represent hydrogen bond interactions of DDX23 and GW4064. **d** Luciferase assay of luc-21-miRDREL-expressing A549 cells treated with compound #23. Derepressed firefly luciferase activity represents the inhibition of miR-21 biogenesis by compound #23 in the luc-21-miRDREL-expressing A549 cells. The data represent the mean values of four independent experiments (*n* = 4). The error bars in the graph represent ±standard deviation, and the *P*-values are based on a comparison of the control (DMSO) to compound #23. ***P < 0.001. **e** Proliferation assay of luc-21-miRDREL-bearing A549 cells treated with compound #23. The data represent the mean values of four independent experiments (*n* = 4). The error bars in the graph represent ±standard deviation. **P < 0.01, ***P < 0.001; RLU, relative luminescent unit. **f** Northern blot analysis of miR-21 expression after PANC-1 cells treatment with ivermectin and compound #23.

As a luminescence system can be eminently suitable for high-throughput screening of oncomiR inhibitor candidates, we examined whether our miRDREL system could be used to help improve the inhibitory and delivery efficacy of ivermectin against brain tumors. To develop a more effective ivermectin-based miR-21 inhibitor, we first analyzed the X-ray crystallographic structure of DDX23, which was obtained from the Protein Data Bank (PDB code; 4NHO). Through molecular modeling, we predicted the binding between ivermectin and DDX23. The predicted docking model suggested that ivermectin would bind to the ATP-binding pocket of DDX23. To identify a new compound that can bind to the ATP-binding pocket of DDX23, we conducted molecular docking-based virtual screening using in-house chemical libraries. Compounds were selected based on their drug properties and docking scores (data not shown). This virtual screening led to the identification of 106 candidate compounds. Then, we assessed the inhibitory efficacy of the sorted compounds against miR-21 processing in luc-21-miRDREL-expressing PANC-1 cells (Fig. 6a). Among the 106 compounds, compounds #11 and #23 showed potent inhibition of miR-21 processing.

Interestingly, compound #23 [GW4064; 3-(2,6-dichlorophenyl)-4-(3′-carboxy-2-chlorostilben-4-yl)oxymethyl-5-isopropylisoxazole, a synthetic agonist of farnesoid X receptor]^46^ was found to be a more potent miR-21 inhibitor than ivermectin, with both located at a similar site in the ATP-binding pocket of DDX23 (comparison of Figs. 6b, c). As expected, luc-21-miRDREL-expressing A549 cells also showed strong dose-dependent induction of luciferase activity following exposure to compound #23 (Fig. 6d). The proliferation rate of luc-21-miRDREL-expressing A549 cells (Fig. 6e) and the expression level of miR-21 in PANC-1 cells (Fig. 6f) were significantly impaired by treatment with compound #23. By comparing the predicted models of DDX23 binding with ivermectin and with compound #23 (GW4064), we were able to predict that hydrogen bonding with Ser439, Gln418, Lys412, and Glu413 may be essential for the inhibition of DDX23 activity by GW4064 (Fig. 6b, c). All the data indicated that GW4064 potentially blocks pri-miR-21 processing by decreasing the activity of DDX23 in a similar mode of action to ivermectin.

### Using miRDREL to identify and target oncogenic kinome-DDX23-miR-21 signaling

As shown in Figs. 5 and 6, the ability of ivermectin and an ivermectin derivative to inhibit oncogenic DDX23-miR-21 signaling was effectively validated with luc-21-miRDREL in vitro and in vivo. As a specific modulator for the expression of miR-21, DDX23 itself would be an attractive target for additional miR-21-based drug development^19^. Previous studies indicated that various microenvironmental stimuli (e.g., cellular kinase signaling) are essential for the action and regulation of miRNAs. Moreover, posttranslational modification (e.g., phosphorylation) of miRNA machinery and microprocessors is critical for the biogenesis and turnover of miRNAs^36^. Considering both previous and present findings, we hypothesized that the positive miR-21-processing activity of DDX23 may be finely tuned by protein–protein interactions (PPIs) (e.g., in collaboration with the Drosha microprocessor) and posttranslational modification.

**Fig. 7:**
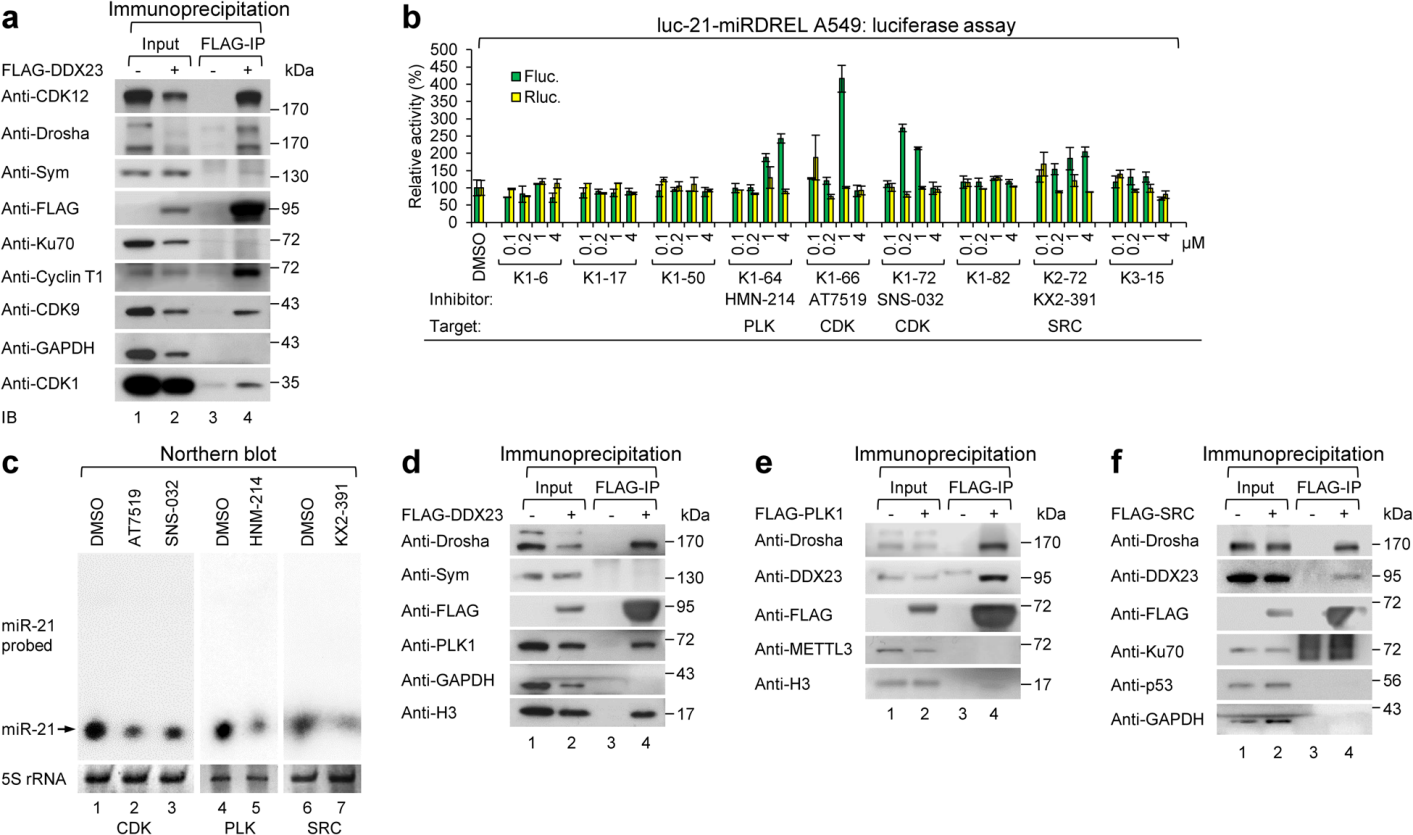
Using miRDRELs to identify and target oncogenic kinome-DDX23-miR-21 signaling. **a** Association of DDX23 with cyclin-dependent kinase (CDK) family members in 293T cells. FLAG-DDX23 was overexpressed in 293T cells and immunoprecipitated with anti-FLAG M2 affinity gels. The precipitates were analyzed by western blotting of anti-CDK family members (anti-CDK1, anti-CDK9, anti-cyclin T1, and anti-CDK12) and anti-Drosha. Symplekin (Sym), Ku70, and GAPDH were also analyzed as negative binding controls. Anti-FLAG represents precipitated FLAG-tagged DDX23 (FLAG-DDX23). **b** Screening of the kinase inhibitor library with luc-21-miRDREL-expressing A549 cells and the identification of new miR-21 biogenesis inhibitors. luc-21-miRDREL-expressing A549 cells were treated with kinase inhibitors (0.1, 0.2, 1, and 4 μM) for 48 h and then assayed for luciferase activity. The firefly luciferase (green bars) and *Renilla* luciferase (yellow bars) activities from the luc-21-miRDREL-expressing A549 cells are presented separately. The data represent the mean values of three independent experiments (*n* = 3). The error bars in the graph represent ±standard deviation. **c** Northern blot analysis of miR-21 expression in the luc-21-miRDREL-expressing A549 cells treated with AT7519 (a CDK inhibitor), SNS-032 (a CDK inhibitor), HNM-214 (a PLK1 inhibitor), or KX2-391 (a SRC inhibitor). The arrow indicates the band of mature miR-21. 5S rRNA was used as a loading control. **d**–**f** Association of DDX23 with PLK1 and SRC in 293T cells. **d** FLAG-DDX23 was overexpressed in 293T cells and immunoprecipitated with anti-FLAG M2 affinity gels. The precipitates were analyzed by western blotting with anti-PLK1 as well as anti-Drosha. Symplekin (Sym) and GAPDH were also analyzed as negative binding controls. **e** FLAG-PLK1 was overexpressed in 293T cells and immunoprecipitated with anti-FLAG M2 affinity gels. The precipitates were analyzed by western blotting with anti-DDX23 as well as anti-Drosha. METTL3 and histone H3 (H3) were also analyzed as negative binding controls. **f** FLAG-SRC was overexpressed in 293T cells and immunoprecipitated with anti-FLAG M2 affinity gels. The precipitates were analyzed by western blotting with anti-DDX23 as well as anti-Drosha. Ku70, p53, and GAPDH were also analyzed as negative binding controls. Anti-FLAG represents precipitated FLAG-DDX23, -PLK1, and -SRC in panels (**d**), (**e**), and (**f**), respectively.

To probe these possibilities, we analyzed PPIs by performing a pull-down assay of DDX23 in cells and analyzing the interactome by liquid chromatography-tandem mass spectrometry (LC-MS/MS). To our surprise, the analysis led to the identification of various cyclin-dependent kinase (CDK) family members, including CDK1, CDK9, cyclin T1 (the heterodimeric partner of CDK9), and CDK12 (data not shown). As shown in Fig. 7a, the interactions between DDX23 and the identified members of the cyclin-dependent kinome were confirmed by FLAG-DDX23 pull-down assay and subsequent western blot analysis.

We next used luc-21-miRDREL-expressing A549 cells to perform high-throughput screening with a kinase inhibitor library (Fig. 7b). Interestingly, the identified CDK inhibitors AT7519 and SNS-032 have broad specificities for various CDKs; AT7519 shows strong inhibitory effect on CDK1 (IC50 = 0.19 µM) and SNS-032 is a potent and specific inhibitor of CDK9 (IC50 = 4 nM) (Supplementary Table 3). Our kinase inhibitor screen also identified inhibitors of polo-like kinase 1 (PLK1; HNM-214) and SRC (KX2-391) as impacting miR-21. This is the first and novel report that these kinases could be involved in regulating miR-21.

The independent regulation of miR-21 biogenesis by the identified kinase inhibitors was also biochemically validated by applying these inhibitors to luc-21-miRDREL-expressing A549 cells and analyzing miR-21 expression by northern blotting. As shown in Fig. 7c, each of the tested inhibitors downregulated the expression of miR-21 in the luc-21-miRDREL-expressing A549 cells.

To identify the potential relationship between the DDX23-Drosha complex and PLK1, we tested their physical association with coimmunoprecipitation assay in 293T cells. As shown in Fig. 7d, PLK1 was coimmunoprecipitated with the DDX23-Drosha complex. Moreover, the reverse associations—those of DDX23-Drosha with PLK1 or SRC—were confirmed by FLAG-PLK1 and FLAG-SRC pull-down assay followed by western blotting (Fig. 7e, f) and LC-MS/MS of FLAG-PLK1 (data not shown).

**Fig. 8:**
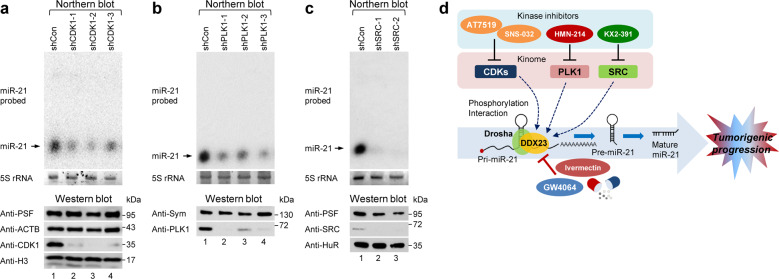
Depletion of identified kinome abrogated the expression of miR-21 and novel targeting strategy for kinome-DDX23-miR-21 signaling during tumorigenesis. **a** Specific knockdown of CDK1 abrogated the expression of miR-21 in luc-21-miRDREL-expressing A549 cells. The cells were infected with lentivirus expressing CDK1-specific shRNAs, and total RNA was prepared and subjected to northern blotting. The level of CDK1 was analyzed by western blotting after specific knockdown of CDK1 in the luc-21-miRDREL-expressing A549 cells. PSF, ACTB, and histone H3 (H3) were also analyzed as negative loading controls (bottom). **b** Specific knockdown of PLK1 abrogated the expression of miR-21 in the luc-21-miRDREL-expressing A549 cells. The cells were infected with lentivirus expressing PLK1-specific shRNAs, and total RNAs was prepared and subjected to northern blotting. The level of PLK1 was analyzed by western blotting after specific knockdown of PLK1 in the luc-21-miRDREL-expressing A549 cells. Symplekin (Sym) was also analyzed as a negative loading control (bottom). **c** Specific knockdown of SRC abrogated the expression of miR-21 in the luc-21-miRDREL-expressing A549 cells. The cells were infected with lentivirus expressing SRC-specific shRNAs, and total RNA was prepared and subjected to northern blotting. The protein level of SRC was analyzed by western blotting after specific knockdown of SRC in the luc-21-miRDREL-expressing A549 cells. PSF and ELAVL1/HuR (HuR) were also analyzed as negative loading controls (bottom). **d** Newly identified miR-21-processing inhibitor GW4064 with the miRDREL system is shown as blue oval. The pharmacological kinase inhibitors that modulate miR-21 processing identified with the miRDREL are also shown.

Furthermore, to test whether miR-21 expression is directly regulated by the kinome identified as being potentially mediated by DDX23-Drosha complex association and phosphorylation, we also generated small hairpin RNAs (shRNAs) for each identified kinase and transduced these constructs into luc-21-miRDREL-expressing A549 cells (Supplementary Table 2). As shown in Fig. 8a–c, northern and western blot analyses demonstrated that the abilities of the identified inhibitors to decrease miR-21 expression were directly mediated by the relevant kinases and potentially via DDX23-Drosha complex association and phosphorylation.

## Discussion

In this study, we developed and intensively applied a sensitive and effective system for monitoring oncomiR action and biogenesis in vitro and in vivo. The specificity and sensitivity of our “miRDREL” system for various oncomiRs were validated by showing that the miRNA expression level was highly correlated with the targeting efficacy of the system in various cancer cell lines (Fig. 1 and Supplementary Figs. 2, 3). Transfection of mimics and depletion of specific oncomiRs with antagomirs further confirmed the specificity of the system (Fig. 1 and Supplementary Fig. 4). The use of miRDRELs bearing natural miRNA target sequences demonstrated that the system is also effective for cellular oncomiR targets (Fig. 2). Moreover, experiments involving depletion of key microprocessors and physiological triggering of oncomiRs demonstrated that the system can be used to monitor the cellular miRNA-processing machinery (Fig. 3). The collective data indicate that the generated miRDREL system is a useful tool in oncomiR-related cancer research.

The data in the latter part of the present report show that our miRDREL system can be used for preclinical and translational purposes in various cellular and small animal contexts in vitro and in vivo (Figs. 4–8). We used a miRDREL to verify ivermectin as an inhibitor of oncogenic DDX23-miR-21 signaling and then investigated molecular modeling-based high-throughput screening to identify its new derivative, GW4064, that has improved action (Fig. 6). We then applied miRDRELs to a detailed screening-based investigation of kinome-DDX23-miR-21 signaling that expanded our understanding of alternative approaches for targeting oncomiRs in cancer therapeutics (Fig. 7). We integrated our experimental results and observations into an oncomiR inhibitory strategy that can be used to specifically block the action of miR-21 in cancer (Fig. 8d). These findings collectively support our contention that the miRDREL system is suitable for basic mechanism-based research and has special merit for preclinical and translational purposes, such as a diagnostics and drug development pipeline for various diseases, especially cancer.

Investigators frequently face difficulty in delivering miRNA-sensing systems into the appropriate target cells. Generally, plasmid-based transient transfection strategies are limited by uneven transfection, low transfection efficiency (such as in terminally differentiated cells of neural origin and nondividing cells), and the need for multiple plasmids for a given assay. Retroviral system delivery is limited to nondividing and primary cells. The multiple cistronic expression of multiple genes and transcriptional interference arising from each reporter can cause additional problems for high-throughput applications. Multiple rounds of infection/transfection and measurements of reporter activity can be nearly impossible in tissues of living animals and patient-related specimens. Frequently, inaccurate normalization of reporter activity complicates the interpretation of data.

To overcome these hurdles, researchers are currently seeking to develop various approaches to sense miRNA action, such as molecular beacon-based systems and nanodelivery strategies. However, these transient delivery approaches are not adequate for long-term in vivo animal experiments or for maintaining system consistency. A new system should show strong stability and persistence to support the achievement of consistent screening results. It should also be easily applied to various model systems (e.g., cancer cell lines, primary cells, animal tissues, and patient specimens). Our system is superior to the aforementioned conventional systems, as indicated by the following lines of evidence.

First, our miRDREL system bears a counter-directed promoter-driven reporter and thus offers the benefit of expressing two desired transgenes (dual luminescence or fluorescence) from a single unit, arranged in opposite directions under the influence of CMV and hPGK promoters in cassette form. Therefore, each reporter transcript is expressed from an amenable promoter without transcriptional disruption. As the primary sensors, a firefly luciferase luminescence reporter and EGFP fluorescence reporter were used to sense the action of the target miRNA in vitro and in vivo; their activities were normalized to the activities of *Renilla* luciferase and mCherry, respectively. The activities of these readouts reflect normal cap-dependent translation in cells from independent opposed cistrons. This strategy avoids the potential problems of performing standardization in conventional single-gene reporter systems. In the miRDREL system context, the dual-luciferase or fluorescence measurement of miRNA-controlled signals is monitored simply and easily in various cellular systems.

Second, our system offers the benefit of enabling the promoter range to be expanded. Depending on the researcher’s purpose, various promoting units can be used to drive sensor transcription in our miRDREL system. Here, we used CMV and hPGK promoters for sensing the miRNA and normalization units, respectively. We also tested the applicability of minimal promoter elements such as hypoxia regulatory element (HRE) repeats for monitoring strong tumorigenic cues, hypoxia (data not shown), the core EF1A sequence (data not shown), and a cell/tissue-specific promoter (e.g., Syn; neuron-specific synapsin 1, data not shown). The use of a tissue-specific promoter in the expression cassette can inhibit unwanted transgene expression and facilitate persistent transgene expression in a specific cellular microenvironment. We also improved the virus titer to a significant degree by inserting axillary regulatory elements (WPRE and cPPT) into the system (data not shown).

Third, our system has the advantage of offering expansion potential in terms of the sensing reporters. For example, to increase the sensitivity of luc-miRDREL, the reporter element can be replaced with NanoLuc luciferase^39^. Moreover, by inserting the naturally secreted *Gaussia* and *Cypridina* luciferases^47^, the system can be used to monitor drug reactivity over time in culture medium without the need to sacrifice cells^48^. Compared to EGFP, these bioluminescence-based sensing reporters can be used to easily and effectively visualize the in vivo function of miRNAs in real-time when cells or animals are pretreated with the relevant substrates, such as *Renilla* or firefly luciferase substrate. Furthermore, as shown in Fig. 5c, our system enables the in vivo dual bioluminescence imaging of drug efficacy in a living mouse xenograft model without the need to sacrifice the animal.

Fourth, our miRDREL system can be easily loaded with various oncomiR targets and RNA *cis*-elements, such as those in a *cis*-element library for RNA sensing. We systematically verified the operation, usefulness, and specificity of our miRDREL system with miRNA-targeting *cis*-elements, including established oncomiRs [miR-21, let-7a, miR-7, miR-122, and miR-210 (data not shown)] and natural miRNA-targeting sites, such as those harbored in mRNAs encoding CDC34, PPP1R3B, SATB1, and GYS1 in various cancer cell lines and in animal tumor models. The presence of versatile multicloning sites in the 5′ and 3′UTRs of the primary sensor (firefly luciferase or EGFP) makes our system more useful because it enables the insertion of, for example, a primary miRNA sequence (data not shown), iron regulatory element, and internal ribosome entry site in the 5′UTR. Potential 3′UTR insertion candidates might include RNA-binding protein regulatory elements (e.g., AU-rich elements), iron regulatory elements, HIV trans-activation response elements, cytoplasmic polyadenylation elements [UUUUUA_1-2_U_2_]^49,50^, and even miRNA sponges^51^.

In this study, we showed that the miRDREL system can be used to efficiently and precisely monitor oncomiR action throughout long-term experiments conducted in small animals. This long-term measurement ability enables researchers to more fully grasp the effect of the microenvironment on tumor formation and malignancy, such as inflammatory signaling, hypoxia, and recurrence of cancer. Moreover, our sensors consistently yielded results that were highly reliable, reproducible, and reflective of the in vivo situation.

The final major advantage of our system is its ability to be optimized for drug candidate validation and high-throughput screening. In vitro, a luminescence system is considered to be the most suitable for high-throughput screening of candidate oncomiR inhibitors. A few reports have described drug development approaches that have involved miRNA targeting. For example, the antibacterial drug enoxacin was shown to inhibit tumor growth by modulating TARBP but also reportedly exhibited broad-spectrum regulation of overall miRNA processing^52^. However, in certain cases, the miRNA inhibitor candidates screened were not appropriate for translation to the clinic due to potential issues with toxicity and/or side effects. Our group and others previously showed that miR-21 expression is closely correlated with the malignancy of various cancers^19,22,23,42–44^; it is thus generally considered to act as an oncogene by negatively regulating various tumor-suppressive targets. A strategy aimed at inhibiting the expression and biogenesis of miR-21 may therefore be valuable for managing tumor malignancy.

Using the miRDREL system, we herein confirmed the miR-21-inhibiting action previously identified for the drug ivermectin. As shown in Fig. 5, we then expanded the usage of the miRDREL system to fields of preclinical and basic research by using it to deepen our understanding of the ivermectin-DDX23 axis for miR-21 signaling, both in vitro and in an animal model in vivo. We also verified that miRDRELs can be used for quantifying and visualizing the action of ivermectin on miR-21 processing in small animals in vivo.

Mechanism-based elucidation of miRNA action is critical since the identified chemical (e.g., ivermectin) can be easily modified based on target-oncomiR cross talk and catalysis. In this respect, DDX23 and the specific miR-21-processing inhibitor ivermectin appear to form a very attractive molecular pipeline for miRNA-related drug development. However, ivermectin has a macrocyclic lactone structure with olefin bonds composed of 22 and 23 carbons, and it is composed of butyl and ether groups, limiting its ability to penetrate deep into human organs or cross the blood–brain barrier to reach the brain, where RNA helicase DDX23-miR-21 signaling takes place. We thus set out to identify a new relevant derivative of ivermectin. We performed systemic molecular modeling and biochemical analysis based on the molecular docking structure of ivermectin-DDX23. From this, we newly identified GW4064, which has a molecular docking strategy similar to ivermectin, as a new miR-21-processing inhibitor (Fig. 6).

Our miRDREL-based analysis of the ivermectin-DDX23-miR-21 axis expands our understanding of the potential kinomic regulation during miR-21 signaling in the context of both preclinical and basic mechanistic studies. The contribution of cellular kinases to oncomiR signaling has not been studied in detail and warrants further investigation. As shown in Fig. 7, our pull-down experiments and LC-MS/MS data suggest that the phosphorylation of the DDX23-Drosha complex may modulate miR-21 processing. The implication that various CDKs, including CDK1, CDK9, cyclin T1, and CDK12, are involved in this process suggests that miR-21 processing has an upstream kinase pathway that can be regulated by the posttranslational modification of its specific modulator, DDX23. Notably, the phosphorylation of three serine residues (Ser106, Ser107, and Ser109) at the N-terminus of DDX23 is greatly enhanced by nocodazole treatment, which arrests cells in G_2_/M and leads to the highest CDK1 activity^53^. Moreover, essential observations of RNA regulation suggest that CDK9/cyclin T1 may be involved in DDX23-mediated miR-21 processing^54,55^.

As shown in Fig. 7b, additional high-throughput screening strongly suggested that cellular kinase signaling contributes to the modulation of DDX23 activity in the context of miR-21 biogenesis. Identification of CDK inhibitors can be predicted; however, the identification of PLK1 and SRC inhibitors suggests that the signaling involves multiple factors and is more complicated than we expected. Intriguingly, the identified DDX23-interacting kinome and the screened kinase inhibitors for CDKs, PLK1, and SRC, seem likely to be involved in the fine-tuned regulation of miR-21 processing. Specific kinome-mediated phosphorylation may change the catalytic activity of the DDX23-Drosha complex to increase miR-21 expression at the posttranscriptional level in a tumorigenic environment. This supposition suggests that the involved kinase signaling may be another attractive route for the development of miR-21-targeting drugs.

The specificity of the screened inhibitors for miR-21 expression was additionally validated by northern blotting, which is the most widely used quantitative biochemical analysis after cell treatment with identified inhibitors (Fig. 7c). Moreover, as shown in Fig. 8a–c, the postulated ability of each kinase to regulate miR-21 processing was confirmed by specific knockdown and consecutive northern blot analysis. The obtained data clearly demonstrate that the identified cellular kinases are genuine modulators of the biogenesis of miR-21.

The data obtained from our independent experimental approaches and observations were integrated into a single plausible pathway, which is shown in the model presented in Fig. 8d. We verified this integrated pathway through approaches based on the signaling mode (kinome-DDX23-miR-21 axis) and assessed pharmaceutical and preclinical aspects (miR-21 inhibitors and animal models).

We herein report the development and systemic preclinical application of a sensitive and effective system for functional measurement and monitoring of oncomiR actions, essentially miR-21, in vitro and in vivo. The developed miRDREL system is an effective and powerful platform for cancer therapeutics involving prognostic marker and inhibitor screening based on the efficient sensing of oncomiRs. We think that the system will not only be useful for basic laboratories interested in miRNA biology, but also more broadly in the translational and clinical approaches for monitoring the actions of oncomiRs in cancer.

## Supplementary information

Supplemental Material
