# Supplementary material for: Development of a miRNA-controlled dual-sensing system and its application for targeting miR-21 signaling in tumorigenesis
Source: Exp Mol Med. 2020 Dec 11;52(12):1989–2004. doi: 10.1038/s12276-020-00537-z (PMC8080684; doi:10.1038/s12276-020-00537-z)
Supplement: Supplementary file 1 — Supplemental Material [file 12276_2020_537_MOESM1_ESM.pdf]

Supplementary Information

Development of a miRNA-controlled dual-sensing system and its application for targeting miR-21 signaling in tumorigenesis

Yoona Seo[1,2], Sung Soo Kim[1,3], Namdoo Kim[5], Sungchan Cho[4], Jong Bae Park[1,3], and Jong Heon Kim[1,2]

[1] Department of Cancer Biomedical Science, Graduate School of Cancer Science and Policy, National Cancer Center, Goyang 10408, Republic of Korea. [2] Division of Cancer Biology, Research Institute, National Cancer Center, Goyang 10408, Republic of Korea. [3] Division of Clinical Research, Research Institute, National Cancer Center, Goyang 10408, Republic of Korea. [4] Targeted Medicine Research Center, Korea Research Institute of Bioscience and Biotechnology, Ochang 28116, Republic of Korea. [5] Voronoi Bio Inc., Incheon, 21984, Republic of Korea

Correspondence: Jong Heon Kim (jhkim@ncc.re.kr)

[1] Department of Cancer Biomedical Science, Graduate School of Cancer Science and Policy, National Cancer Center, Goyang 10408, Republic of Korea

[2] Division of Cancer Biology, Research Institute, National Cancer Center, Goyang 10408, Republic of Korea.

Table of Contents

Supplementary Results……………………………………………………………………3

Supplementary Materials and methods……………………………………………………4

Plasmids………………………………………………………………………………………4

Lentivirus production, infection, and establishment of stable cell lines……………………..5

Transient transfection of RNA oligomers…………………………………………………6

Fluorescence detection of miRDREL *in vitro* and *in vivo*………………………………...6

Molecular modeling…………………………………………………………………………7

Transient transfection of plasmids and immunoprecipitation……………………………..7

Antibodies and western blotting………………………………………………………………7

Supplementary Tables……………………………………………………………………...9
Supplementary Figures…………………………………………………………………14
Supplementary References………………………………………………............................22

Supplementary Results

Development of miRDRELs, validation of dual-promoter activity, and miRNA-targeting efficacy

To design the efficient miRNA dual-sensing system considering several aspects including dual-promoter action and miRNA targeting efficacy, first, we coordinated the promoters to enable the efficient transcription of the miRNA-targeting site bearing main sensor in its 3′UTR and the normalization readout nearby (Supplementary Fig. 1). Various promoters were carefully tested for transcription of the main sensor (data not shown); the cytomegalovirus (CMV) immediate-early promoter, which is widely used for reporter transcription [1], was primarily installed as the basic promoter for the miRNA-sensing reporter. As the promoter for the normalization reporter, we chose the human phosphoglycerate kinase 1 promoter (hPGK), which is commonly used in lentiviral vectors. It showed relatively constant activity and no interference with the adjacent promoter [2-5]. As shown in Supplementary Fig. 1b-c, the normalization promoter was arranged in the opposite direction relative to the readout promoter to avoid potential transcriptional readthrough within same transcriptional orientation. A spacer of 27 nucleotides (nt) was also inserted between the two adjacent promoters to avoid potential interference [6].

As the primary reporters for sensing miRNA actions *in vitro* and *in vivo*, firefly luciferase and enhancer green fluorescence protein (EGFP) were used [7]. Normalization of firefly luciferase or EGFP for the sensing miRNA action can be performed using *Renilla* luciferase or a member of the mFruits family of monomeric red fluorescent proteins (mCherry) [8], respectively. In all relevant constructs, the various miRNA-targeting *cis*-elements (including the target-site-null control) were inserted into the 3′UTR of the firefly luciferase or EGFP reporters (Supplementary Fig. 1).

Next, a sequence perfectly complementary to the miR-21 target site was inserted with various repeat numbers near the 3′ end of a firefly luciferase reporter open reading frame. Constructs bearing various numbers of miR-21 target sites were generated (1, 2, 3, 4, 5, 6, 7, 8, and 12 × repeats) and the translational repression efficiency was examined by transiently transfecting the constructs into human hepatocellular carcinoma Huh7 cells. As shown in Supplementary Fig. 2, the translational repression rate showed a relatively linear correlation with the number of targeting sites. The miR-21 targeting sensors seemed to be largely divided into two groups (group I: 1, 2, 3 and 4 × repeats; group II: 5, 6, 7, 8 and 12 × repeats) depending on repression degree of firefly luciferase activities. Considering the synthetic cost and nucleotide accuracy of longer-length oligomers, we chose five tandem repeats as the representative number of miRNA target for the miRDRELs.

Supplementary Materials and methods

Plasmids

luc-Con-miRDREL (luc-Con-miRDREL-I) was constructed as follows: pLJ (J.H.K., unpublished plasmid) which is the derivative of pLenti6/V5-DEST (Invitrogen, Waltham, MA, USA), the gateway entry region was excised with EcoR I-Xho I. Pair of complementary oligomers (sense, 5′-AATTCGCTAGCTGCAGTCGACTGTACAC-3′ and antisense, 5′-TCGAGTGTACAGTCGACTGCAGCTAGCG-3′) were annealed in 1× annealing buffer [0.1 M NaCl, 10 mM Tris-HCl (pH 7.5)]. After incubation at 95°C for 5 min, oligomers slowly cooled down by turning off the heat block. Annealed oligomers were inserted into the EcoR I-Xho I digested pLenti6/V5-DEST for generating pLJ. For the generation of pLJ-Fluc, the firefly open reading frame (ORF) was amplified with pGL3-con (Promega, Madison, WI, USA) and the following oligomers; sense, 5′-CGACTCTAGAGGATCCGCCACCATGGAAGACGCC-3′ and antisense, 5′-ACCACACTGGACTAGTTTACACGGCGATCTTTCCG-3′. 1.5-kb PCR product was digested with Dpn I and gel purified. Purified PCR fragment was inserted into BamH I and Spe I digested pLJ with In-Fusion HD cloning kit (Takara Bio Inc., Shiga, Japan). luc-miRDREL was constructed by insertion of two DNA fragments into Kfl I-Cla I digested pLJ-Fluc with In-Fusion HD cloning kit. The DNA fragment information as follows: DNA fragment 1 (*Renilla* luciferase) was amplified from pRL-TK (Promega) with following oligomers; sense, 5′-CTCCCCAGGGGGATCTAATACGACTCACTATAGGCTAG-3′ and antisense, 5′-GGATCTCGACGGTATCGAATTATTGTTCATTTTTGAGAACTCG-3′. DNA fragment 2 (hPGK promoter) was amplified from pLKO.1puro (Addgene, Watertown, MA, USA) with following oligomers; sense, 5′-AACTCCCAAGCTTATCGATTCGAGGGGGTTGGGGTTG-3′ antisense, 5′-GATCCCCCTGGGGAGAGAG-3′. For the construction of luc-Con-miRDREL-II [luc-Con-miRDREL_(-) Nhe I], luc-21-miRDREL, luc-let-7a-miRDREL, luc-7-miRDREL, luc-122-miRDREL, luc-CDC34-2×21-miRDREL, luc-GYS-2×122-miRDREL, luc-PPP1R3B-2×21-miRDREL, and luc-SATB1-2×21-miRDREL, pair of complementary oligomers (see Supplementary Table 1) were annealed and inserted into the EcoR I-Xho I excised luc-miRDREL. For the generation of mCherry-Fluc-21-miRDREL (J.H.K., unpublished plasmid) which is the intermediate construct of fluo-21-miRDREL, mCherry ORF was amplified from pmCherryC1 [9] with following oligomers: sense, 5′-CTCACTATAGGCTAGCCACCATGGTGAGCAAGG-3′ and antisense, 5′-CAACCCCGAGGGGACCCTTACTTGTACAGCTCGTCCATGC-3′. Purified PCR fragment was inserted into Kfl I-Nhe I digested luc-21-miRDREL with In-Fusion HD cloning kit. For the

construction of fluo-21-miRDREL, EGFP ORF was amplified from pLL3.7 (Addgene) with following oligomers: sense, 5′-CGGGATCCGCCACCATGGTGAGCAAG-3′ and antisense, 5′-GGAATTCCACCACACTGGACTAGTCTACTTGTACAGCTCGTCC-3′. Purified PCR fragment was ligated with EcoR I-BamH I digested mCherry-Fluc-21-miRDREL. For the generation of fluo-Con-miRDREL (fluo-Con-miRDREL-I) which is miRNA targeting site null control construct, pair of complementary oligomers (sense, 5′-AATTCGCTAGCTGCAGTCGACTGTACAC-3′ and antisense, 5′-TCGAGTGTACAGTCGACTGCAGCTAGCG-3′) were annealed and inserted into EcoR I-Xho I digested fluo-21-miRDREL. For the construction of fluo-Con-miRDREL-II [fluo-Con-miRDREL_(-) Nhe I], and fluo-let-7a-miRDREL pair of complementary oligomers (see Supplementary Table 2) were annealed and inserted into the EcoR I-Xho I digested fluo-miRDREL. 3×, 6×, 7×, 8× and 12× repeats of various miR-21 target sites bearing pcDNA3-Fluc constructs were obtained during pcDNA3-Fluc-5×miR-21 construction by manipulation of oligomer annealing. For the generation of pcDNA3-FLAG-PLK1, human PLK1 was amplified from the pEGFPc1-hPLK1 (J.H.K., unpublished plasmid) with following oligomers; sense, 5′-AGGCGGCCGCGGATCCAATGAGTGCTGCAGTGAC-3′ and antisense, 5′-TACCGAGCTCGGATCCTTAGGAGGCCTTGAGAC-3′. PCR product was inserted into BamH I digested pcDNA3-FLAG [10] with In-Fusion HD cloning kit. For the generation of pcDNA3-FLAG-SRC, PCR was assessed with human embryonic kidney 293 cell cDNA and following oligomers; sense, 5′-AGGCGGCCGCGGATCCCATGGGTAGCAACAAGAGC-3′ and antisense, 5′-TACCGAGCTCGGATCCTGTGCCTAGAGGTTCTCC-3′. PCR product was inserted into BamH I digested pcDNA3-FLAG with In-Fusion HD cloning kit. For the generation small hairpin RNA expressing lentiviral constructs for silencing PLK1, CDK1, and SRC, complementary oligonucleotides (Supplementary Table 2) were annealed, and then inserted into the pLKO.1 puro (Addgene) which was digested with Age I-EcoR I. Construction procedures of pFLAG-CMV2-DDX23 was described [11]. All oligomers were purchased from Macrogen (Seoul, Republic of Korea) or Bionics (Seoul, Republic of Korea). All constructs were verified by DNA sequencing (CosmoGenetech, Seoul, Republic of Korea).

Lentivirus production, infection, and establishment of stable cell lines

Lentivirus production was performed as previously reported. Briefly, ~1.5×10^6 of 293FT, 293T, or Lenti-X 293T cells were plated on 100 mm culture dish 48 h before transfection. Then, 4.5 μg of lentiviral construct, 3 μg of psPAX2 (Addgene), and 1.5 μg of pMD2.G (Addgene) were co-transfected into prepared cells using 27 μl of Lipofectamine 2000 (Thermo Fisher Scientific, Waltham, MA, USA) or Metafectene PRO (Biontex, Munich, Germany) in OPTI-MEM (Thermo Fisher

Scientific). The medium was changed with 5 ml culture media without antibiotics 5 h after transfection. After 24 h, caffeine (final concentration, 2 mM) was supplemented in each plate, and then the medium containing lentivirus was harvested 48 h after transfection. Viral particles containing media were filtered through 0.45 μm Acrodisc Syringe Filters with Supor Membrane (Pall, Port Washington, NY, USA), and concentrated using Lenti-X concentrator (Takara Bio Inc.). For the lentivirus infection, 2 × 10^5 of cells were plated on 35 mm culture dishes 48 h before infection and half of purified lentivirus were added onto the target cells in the presence of 10 μg/ml polybrene (Sigma-Aldrich, St. Louis, MO, USA). After incubating for 12~24 h, target cells were recovered 9~24 h with changing complete medium, lentivirus transduced cells were selected with blasticidin S (5 μg/ml; Invivogen, San Diego, CA, USA) or puromycin (2 μg/ml; Enzo Life Sciences, Farmingdale, NY, USA) for 2~3 days.

Transient transfection of RNA oligomers

Synthetic control and miRNA mimics for miR-7, miR-21, and miR-122 were purchased from Bioneer (Daejeon, Republic of Korea) and Ambion (Austin, TX, USA). miRNA inhibitors for miR-21 and its scrambled control were purchased from Integrated DNA Technologies. Small interfering RNAs (siRNAs) were synthesized by Bioneer and Genolution (Seoul, Republic of Korea). siRNA for human Drosha (5′-CAGCAAUGGAUGCGCUUGA-3′), Dicer (5′-AACUCUGCAAACCAGGUUGCU-3′), DDX23 (5′-CCUGUGCAAGUUUGGUGCU-3′), Lin28B (5′-AGGGAAGACACUACAGAAA-3′), and as a negative control, two scrambled siRNAs (5′-ACGUGACACGUUCGGAGAA-3′ and 5′-GUUCAGCGUGUCCGGCGAG-3′) were used for overall knockdown experiments. 4 × 10^4 of luc-miRDREL cells were plated on 24-well cell culture plates 24~48 h before transfection. 20 picomole of siRNA and 1 μl of Lipofectamine 2000 (Thermo Fisher Scientific) were used for each transient transfection. After 48 h, dual luciferase assays were assessed for each luc-miRDREL.

Fluorescence detection of miRDREL *in vitro* and *in vivo*

Fluorescence detection in fluo-miRDREL U87MG cells was carried out through Axio Observer (Zeiss, Oberkochen, Germany). The exhibition of EGFP was compared among cells in different groups, and that of mCherry were used as a control. U87MG mouse model brain tissues EGFP and mCherry fluorescence images were obtained by using an LSM 780 confocal laser-scanning microscope (Zeiss). Nuclei were stained with 4′,6-diamidino-2-phenylindole (DAPI; Sigma-Aldrich).

Molecular modeling

The X-ray crystal structures of DDX23 was retrieved from Protein Data Bank (PDB code; 4NHO). The retrieved protein structure was processed by using Protein Preparation Wizard in Maestro v11.4 software (Schrodinger Release 2017-4: Maestro 11.4, Schrodinger, LLC, New York, NY USA, 2017); addition of all hydrogens, assignment of bond orders, deletion of water molecules. Any missing side chains and missing loops were filled by using prime module integrated within Protein Preparation Wizard. All residues of the proteins were then parameterized using OPLS2005 force field. Finally, restrained minimization was performed until the converged average root mean square deviation (RMSD) of heavy atoms to 0.3 Å. Molecular docking studies of ivermectin and GW4064 on ATP-binding site in DDX23 was carried out using GLIDE module in Schrodinger's package. The best-docked pose was selected as the lowest Glide score.

Transient transfection of plasmids and immunoprecipitation

For the transient transfection of control, pFLAG-CMV2-DDX23, pcDNA3-FLAG-PLK1, and pcDNA3-FLAG-SRC plasmids, 1.5 ~ 3 × 10^6 of 293T cells were plated on poly (L)-lysine (Sigma-Aldrich; 25 μg/ml) coated 100 mm culture dish 24~48 h before transfection. 5 μg of plasmids and 20 μl of METAFECTENE PRO (Biontex) were used for each transient transfection. After 72h, cells were harvested and the overexpression of FLAG-tagged proteins was confirmed by western blotting. Immunoprecipitation (IP) was performed as previously described [10]. Cells were crushed in IP buffer [150 mM NaCl, 25 mM HEPES-KOH (pH 7.5), 10% (v/v) glycerol, 1mM $MgCl_2$, 2 mM sodium orthovanadate, 2 mM β-glycerophosphate, 1 mM phenylmethylsulphonyl fluoride, 1 mM dithiothreitol, 2 mM EDTA, 0.5% Triton X-100 (Sigma-Aldrich) and 1 × protease inhibitor cocktail (Roche, Basel, Switzerland)]. After brief sonication and incubation on ice, lysates were centrifuged at 12,000 × g for 5 min to remove insoluble materials. The lysates were then incubated with anti-FLAG M2 affinity gels (Sigma-Aldrich) for 2 h at 4°C. The collected beads were then washed four times with washing buffer (0.05% Triton X-100 IP buffer without protease inhibitor cocktail) and boiled in SDS gel-loading buffer for western blot analysis.

Antibodies and western blotting

Anti-Nestin (mouse monoclonal, 611658, BD Biosciences, San Jose, CA, USA), anti-EGFR (rabbit polyclonal, sc-03, Santa Cruz Biotech, Santa Cruz, CA, USA), anti-GFAP (mouse monoclonal, clone GA-5, MP Biomedicals, Santa Ana, CA, USA), anti-β-actin (mouse monoclonal, clone C4, Merck Millipore, Burlington, MA, USA; GTX629630, GeneTex, Irvine, CA, USA), anti-GAPDH

(rabbit monoclonal, clone 14C10, Cell Signaling Technology, Danvers, MA, USA), anti-SOX2 (goat polyclonal, AF2018, R&D Systems, Minneapolis, MN, USA), anti-Lin28B (rabbit polyclonal, #4196, Cell Signaling Technology), anti-CDK12 (rabbit polyclonal, #11973, Cell Signaling Technology), anti-Drosha (rabbit polyclonal, ab245398, Abcam, Cambridge, UK; rabbit polyclonal, A301-886A, Bethyl Laboratories, Montgomery, TX, USA), anti-Symplekin (mouse monoclonal, clone 25, BD Biosciences), and anti-FLAG (mouse monoclonal, clone L5, BioLegend, San Diego, CA, USA), anti-Ku70 (mouse monoclonal, A-9, Santa Cruz Biotech), anti-Cyclin T1, (rabbit monoclonal, clone D186G, #81464, Cell Signaling Technology), anti-CDK9 (rabbit monoclonal, clone C12F7, #2316, Cell Signaling Technology), anti-CDK1 (rabbit polyclonal, ab131450, Abcam; mouse monoclonal, SC-54, Santa Cruz Biotech), anti-PLK1 (mouse monoclonal, F-8, Santa Cruz Biotech; rabbit polyclonal, A300-251A, Bethyl Laboratories), anti-Histone H3 (rabbit monoclonal, clone D1H2, #4499, Cell Signaling Technology), anti-DDX23 (rabbit polyclonal, A300-695A, Bethyl Laboratories), anti-METTL3 (rabbit polyclonal, ab66660, Abcam), anti-p53 (rabbit polyclonal, sc-6243, Santa Cruz Biotech), anti-PSF (mouse monoclonal, D-8, Santa Cruz Biotech), anti-SRC (rabbit monoclonal, clone 36D10, #2109, Cell Signaling Technology), and anti-HuR (mouse monoclonal, 3A2, Santa Cruz Biotech) antibodies were used through all the WB analysis. For the WB analysis, whole cell lysate were homogenated with RIPA [50 mM Tris-HCl pH 7.5, 150 mM NaCl, 1% sodium deoxycholate, 0.1% SDS, 1% Triton X-100, 5 mM NaF, 2 mM sodium orthovanadate, 2 mM β-glycerophosphate, 2 mM EDTA, and protease inhibitor cocktail (Roche)], IP buffer, or 1 × passive lysis buffer (Promega) were mixed with SDS gel-loading buffer, denatured for 5 min at 95°C, and loaded onto an 11% polyacrylamide-SDS gel or Novex 4-20% Tris-Glycine Mini Gels (Thermo Fisher Scientific). After electrophoresis, proteins were transferred to a nitrocellulose membrane (Pall) or PVDF (Merck Millipore). Membranes were blocked at room temperature 5% nonfat dry milk in tris-buffered saline (TBS) (50 mM Tris-HCI [pH7.4] and 150 mM NaCl) with 0.1% TWEEN 20 (Amresco, Solon, OH, USA), and then incubated overnight at 4°C with primary antibodies. Membranes were washed three times for 5 min with 1 × TBST prior to incubating with secondary antibodies. As a secondary antibody, horseradish peroxidase-conjugated anti-rabbit (Vector Laboratories, Burlingame, CA, USA), anti-mouse IgG (Vector Laboratories), and anti-rat IgG (Santa Cruz Biotech) were used. Enhanced chemiluminescence signal were detected and quantified by C-DIGIT (LI-COR, Lincoln, NE, USA) and/or X-ray film (AGFA, Mortsel, Belgium) exposure.

Supplementary Tables

Supplementary Table 1. Oligonucleotides used in miRNA target sequence in miRDREL constructs. S, sense; AS, antisense

Plasmid constructs	Sequence of oligonucleotides
luc-Con-miRDREL-II	S: 5′-AATTCGTTAACTGCAGTCGACTGTACAC-3′<br>AS: 5′-TCGAGTGTACAGTCGACTGCAGTTAACG-3′
CDC34_2×let-7	S: 5′-AATTCAGAATAAACTTGCCGAGTTTACCTCACTAGGGCCGGACC CGTGGCTCCTTAGACGACAGACTACCTCACGGAGGTC-3′<br>AS: 5′-TCGAGACCTCCGTGAGGTAGTCTGTCGTCTAAGGAGCCACGGG TCCGGCCCTAGTGAGGTAAACTCGGCAAGTTTATTCTG-3′
GYS1_2×miR-122	S:5′-AATTCCCACCCAGTCCGCCAAACACTCCACCCCCTCCAGCTCCAG TTTCCAAGTTCCTGCACTCCAGAATCCAC-3'<br>AS:5′-TCGAGTGGATTCTGGAGTGCAGGAACTTGGAAACTGGAGCTGG AGGGGGTGGAGTGTTTGGCGGACTGGGTGGG-3'
PPP1R3B_2×miR-21	S: 5′-AATTTGCAAACTGGGTACAATAAATAAGCTGGATTATACAAAGA TATTTAATAAATAAGCTTTCAGTATC-3'<br>AS: 5′-TCGAGATACTGAAAGCTTATTTATTAAATATCTTTGTATAATC CAGCTTATTTATTGTACCCAGTTTGCA-3'
SATB1_2×miR-21	S: 5′-AATTCCAAGCAATTCATACTATAAGCTTCACTCTTATTGTTGTA TGCAATTTTTACTATCATGCAAATAAGCTTC-3'<br>AS: 5′-AATTCCAAGCAATTCATACTATAAGCTTCACTCTTATTGTTGT ATGCAATTTTTACTATCATGCAAATAAGCTTC-3'
1×miR-21_target	S: 5′-AATTCTGTCAACATCAGTCTGATAAGCTAC-3'<br>AS: 5′-TCGAGTAGCTTATCAGACTGATGTTGACAG-3'
2×miR-21_target	S: 5′-AATTCTGTCAACATCAGTCTGATAAGCTATGTCAACATCAGTCT GATAAGCTAC-3'<br>AS: 5′-TCGAGTAGCTTATCAGACTGATGTTGACATAGCTTATCAGACT GATGTTGACAG-3'
4×miR-21_target	S: 5′-AATTCTGTCAACATCAGTCTGATAAGCTATGTCAACATCAGTCT GATAAGCTATGTCAACATCAGTCTGATAAGCTATGTCAACATCAGTCTGA TAAGCTAC-3'<br>AS: 5′-TCGAGTAGCTTATCAGACTGATGTTGACATAGCTTATCAGACT GATGTTGACATAGCTTATCAGACTGATGTTGACATAGCTTATCAGACTGA TGTTGACAG-3'

5×miR-21_target	S: 5′-AATTCTGTCAACATCAGTCTGATAAGCTATGTCAACATCAGTCT<br>GATAAGCTATGTCAACATCAGTCTGATAAGCTATGTCAACATCAGTCTGA<br>TAAGCTATGTCAACATCAGTCTGATAAGCTAC-3'<br>AS: 5′-TCGAGTAGCTTATCAGACTGATGTTGACATAGCTTATCAGACT<br>GATGTTGACATAGCTTATCAGACTGATGTTGACATAGCTTATCAGACTGA<br>TGTTGACATAGCTTATCAGACTGATGTTGACAG-3'
5×let-7a_target	S: 5′-AATTCTTAACTATACAACCTACTACCTCATTAACTATACAACCT<br>ACTACCTCATTAACTATACAACCTACTACCTCATTAACTATACAACCTAC<br>TACCTCATTAACTATACAACCTACTACCTCAC-3'<br>AS: 5′-TCGAGTGAGGTAGTAGGTTGTATAGTTAATGAGGTAGTAGGTT<br>GTATAGTTAATGAGGTAGTAGGTTGTATAGTTAATGAGGTAGTAGGTTGT<br>ATAGTTAATGAGGTAGTAGGTTGTATAGTTAAG-3'
5×miR-7_target	S: 5′-AATTCAACAACAAAATCACTAGTCTTCCAAACAACAAAATCACT<br>AGTCTTCCAAACAACAAAATCACTAGTCTTCCAAACAACAAAATCACTAG<br>TCTTCCAAACAACAAAATCACTAGTCTTCCAC-3'<br>AS: 5′-TCGAGTGGAAGACTAGTGATTTTGTTGTTTGGAAGACTAGTGA<br>TTTTGTTGTTTGGAAGACTAGTGATTTTGTTGTTTGGAAGACTAGTGATT<br>TTGTTGTTTGGAAGACTAGTGATTTTGTTGTTG-3'
5×miR-122_target	S: 5′-AATTCAACAAACACCATTGTCACACTCCAAACAAACACCATTGT<br>CACACTCCAAACAAACACCATTGTCACACTCCAAACAAACACCATTGTCA<br>CACTCCAAACAAACACCATTGTCACACTCCAC-3'<br>AS: 5′-TCGAGTGGAGTGTGACAATGGTGTTTGTTTGGAGTGTGACAAT<br>GGTGTTTGTTTGGAGTGTGACAATGGTGTTTGTTTGGAGTGTGACAATGG<br>TGTTTGTTTGGAGTGTGACAATGGTGTTTGTTG-3'

Supplementary Table 2. Oligonucleotides used in small hairpin RNA (shRNA)-expressing lentiviral constructs. S, sense; AS, antisense

Plasmid constructs	Sequence of oligonucleotides
pLKO.1puro -shPLK1-1	S: 5′-CCG GCC CGA GGT GCT GAG CAA GAA ACT CGA GTT TCT TGC TCA GCA CCT CGG GTT TTT G-3′<br>AS: 5′-AAT TCA AAA ACC CGA GGT GCT GAG CAA GAA ACT CGA GTT TCT TGC TCA GCA CCT CGG G-3′
pLKO.1puro -shPLK1-2	S: 5′-CCG GGC TCA TCT TGT GCC CAC TGA TCT CGA GAT CAG TGG GCA CAA GAT GAG CTT TTT G-3′<br>AS: 5′-AAT TCA AAA AGC TCA TCT TGT GCC CAC TGA TCT CGA GAT CAG TGG GCA CAA GAT GAG C-3′
pLKO.1puro -shPLK1-3	S: 5′-CCG GGT GTG GGT TCT ACA GCC TTG TCT CGA GAC AAG GCT GTA GAA CCC ACA CTT TTT G-3′<br>AS: 5′- AAT TCA AAA AGT GTG GGT TCT ACA GCC TTG TCT CGA GAC AAG GCT GTA GAA CCC ACA C-3′
pLKO.1puro -shCDK1-1	S: 5′-CCG GTG GCT TGG ATT TGC TCT CGA ACT CGA GTT CGA GAG CAA ATC CAA GCC ATT TTT-3′<br>AS: 5′-AAT TAA AAA TGG CTT GGA TTT GCT CTC GAA CTC GAG TTC GAG AGC AAA TCC AAG CCA-3′
pLKO.1puro -shCDK1-2	S: 5′-CCG GGT GGA ATC TTT ACA GGA CTA TCT CGA GAT AGT CCT GTA AAG ATT CCA CTT TTT-3′<br>AS: 5′-AAT TAA AAA GTG GAA TCT TTA CAG GAC TAT CTC GAG ATA GTC CTG TAA AGA TTC CAC-3′
pLKO.1puro -shCDK1-3	S: 5′-CCG GGC TGT ACT TCG TCT TCT AAT TCT CGA GAA TTA GAA GAC GAA GTA CAG CTT TTT-3′<br>AS: 5′-AAT TAA AAA GCT GTA CTT CGT CTT CTA ATT CTC GAG AAT TAG AAG ACG AAG TAC AGC-3′
pLKO.1puro -shSRC-1	S: 5′-CCG GGC TCG GCT CAT TGA AGA CAA TCT CGA GAT TGT CTT CAA TGA GCC GAG CTT TTT G-3′<br>AS: 5′-AAT TCA AAA AGC TCG GCT CAT TGA AGA CAA TCT CGA GAT TGT CTT CAA TGA GCC GAG C-3′
pLKO.1puro -shSRC-2	S: 5′-CCG GCA TCC TCA GGA ACC AAC AAT TCT CGA GAA TTG TTG GTT CCT GAG GAT GTT TTT TG-3′<br>AS: 5′-AAT TCA AAA AAC ATC CTC AGG AAC CAA CAA TTC TCG AGA ATT GTT GGT TCC TGA GGA TG-3′

pLKO.1puro -shScramble (pLKO.1puro -shCon)	S: 5′-CCG GCC TAA GGT TAA GTC GCC CTC GCT CGA GCG AGG GCG ACT TAA CCT TAG GTT TTT G-3′<br>AS: 5′-AAT TCA AAA ACC TAA GGT TAA GTC GCC CTC GCT CGA GCG AGG GCG ACT TAA CCT TAG G-3′

Supplementary Table 3. Brief description of identified kinase inhibitors.

Plate	No.	Item Name	M.W.	Cas #	Target	Selleckchem Catalog #	Brief Description
A	64	HMN-214	424.47	173529-46-9	PLK	S1485	HMN-214 (a prodrug of HMN-176) is a potent PLK1 inhibitor (an average cell IC50 of 118 nM).
A	66	AT7519	382.24	844442-38-2	CDK	S1524	AT7519 is a kinase inhibitor with IC50 of 0.19, 0.044, 0.51, 0.067, 0.66 and 0.018 μM for CDK1/cyclin B, CDK2/cyclin A, CDK2/cyclin E, CDk4/cyclin D1, CDK6/cyclin D3, CDK5/p35.
A	72	SNS-032 (BMS-387032)	380.53	345627-80-7	CDK	S1145	SNS-032 (BMS-387032) is a potent and selective cyclin-dependent kinases (CDK) 9, 7 and 2 inhibitor with IC50 of 4, 62 and 38 nM for CDK9, CDK2/cyclin A and CDK7/cyclin H.
B	72	KX2-391	431.53	897016-82-9	SRC	S2700	KX2-391 is a synthetic, orally bioavailable small molecule and non-ATP competitive SRC tyrosine kinase signaling inhibitor with an IC50 of average 72 nM.

Supplementary Figures

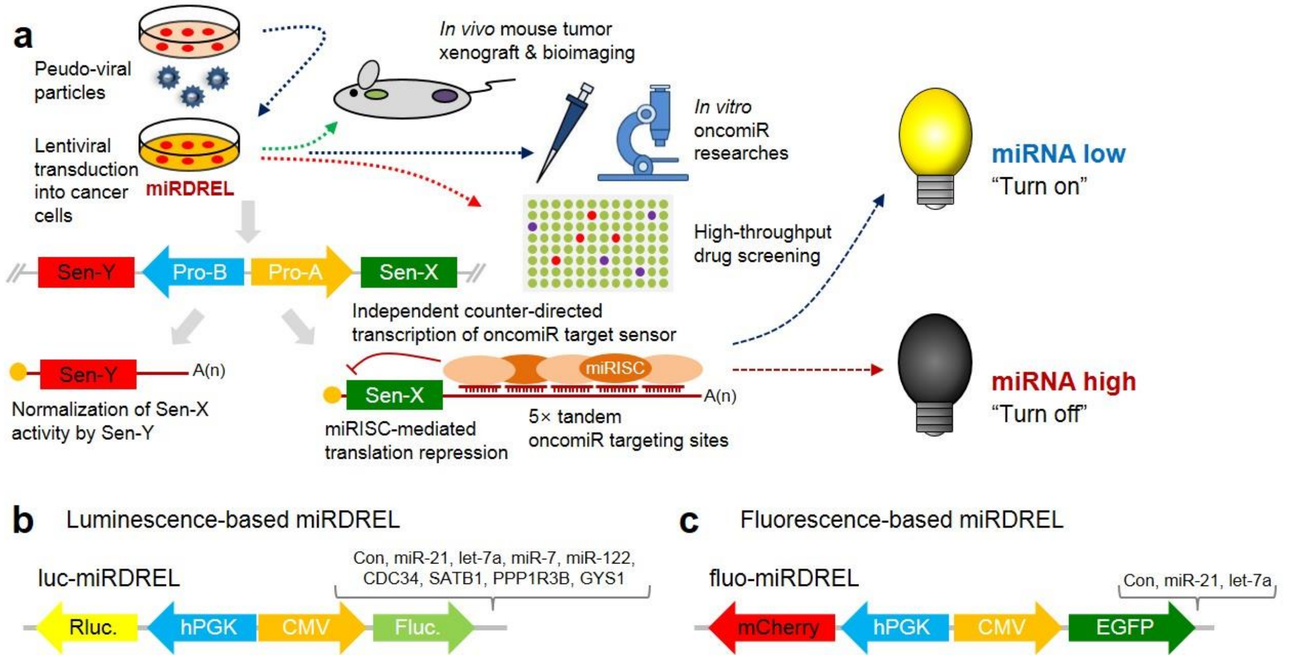

Supplementary Fig. 1 Development of miRDRELs. a Flow chart and schematic diagram of miRDREL generation and its systemic application to the *in vivo* mouse tumor model, bioimaging, high-throughput drug screening, and *in vitro* basic researches for miRNA-sensing. Promoter A (Pro-A) directs expression of five consecutive miRNA-target bearing luminescence (firefly luciferase) and fluorescence (enhanced green fluorescence protein; EGFP) sensors (Sen-X) in those 3′ untranslated region (3′UTR). Promoter B (Pro-B) located in the opposite direction directs luminescence (*Renilla* luciferase) and fluorescence (mCherry) sensors (Sen-Y) which were used as normalization for the miRNA sensor activities which are transcribed from Pro-A. miRISC-meditated translational repression and/or deadenylation of miRNA sensor transcript is dependent on the designated miRNA abundance in cells or tissues. **b** Schematic depiction of the luminescence-based miRDRELs (luc-miRDRELs). Switchable unit Pro-A can be replaced as various units including cytomegalovirus (CMV) which operate miRNA sensors in various cellular environments. Likewise, human phosphoglycerate kinase (hPGK) promoter is basically installed in an opposite direction for directing transcription of normalization sensor (*Renilla* luciferase). Various miRNA target sequences were inserted in the 3′UTR of miRNA sensing luminescence reporter, firefly luciferase. Irrelevant, non-targeting sequence of miRNA was inserted into the control constructs (Con). **c** Schematic depiction of the fluorescence-based miRDRELs (fluo-miRDRELs). Switchable Pro-A can be replaced as various units including

cytomegalovirus (CMV) which operate miRNA sensors in various cellular environments. Likewise, human phosphoglycerate kinase (hPGK) promoter is basically installed in an opposite direction for directing transcription of normalization sensor (mCherry). Various miRNA target sequences were inserted in the 3′UTR of miRNA sensing fluorescence reporter EGFP.

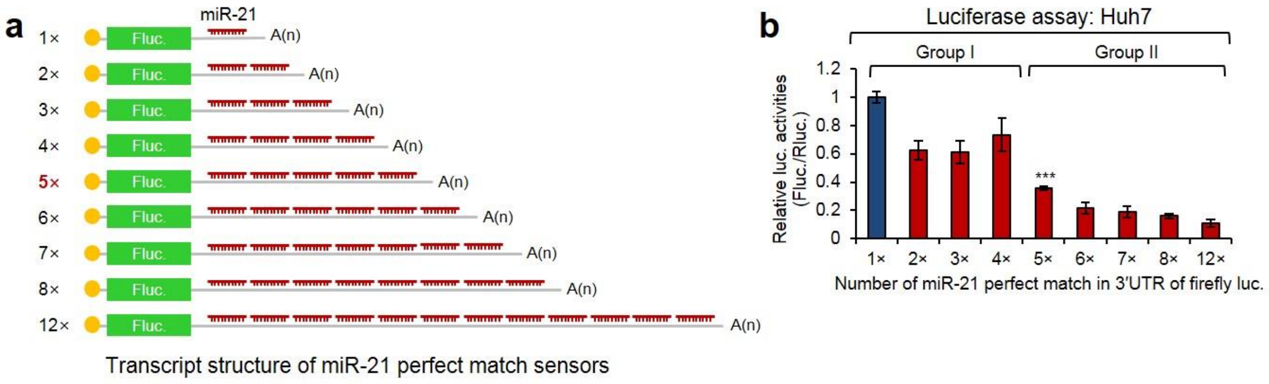

Supplementary Fig. 2 Optimization of target site number for the miR-21 sensor. a Schematic depiction of miR-21 firefly luciferase sensor with various number of miR-21 target sites. **b** Various miR-21 sensors were transiently transfected into the Huh7 cells with normalization control *Renilla* luciferase expressing pRL-CMV plasmid together. After 48 h luciferase activity were measured. Data represent the mean values of three independent experiments ($n = 3$). Error bars in the graph represent ± standard deviation. ***$P < 0.001$.

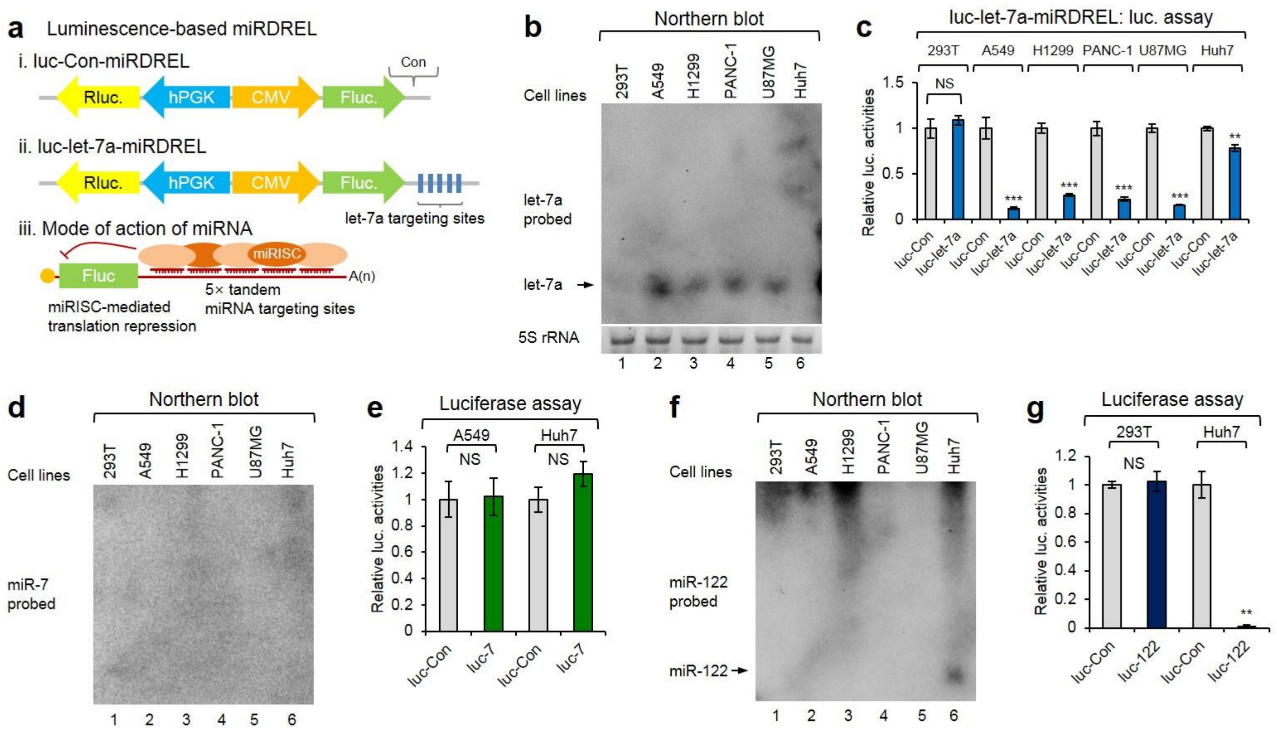

Supplementary Fig. 3 MiRNA-targeting ability of luc-let-7a-miRDREL, luc-7-miRDREL and luc-122-miRDREL in cells. a Schematic depiction of luc-Con-miRDREL (i), luc-let-7a-miRDREL (ii), and mode of action of miRNA targeting to the miRDREL (iii). miRISC; miRNA-induced silencing complex. **b** Northern blot analysis of endogenous let-7a miRNA in various cell lines including human embryonic kidney (293T), non-small cell lung cancer (A549), metastatic lung cancer (NCI-H1299), pancreatic ductal adenocarcinoma (PANC-1), glioblastoma multiforme (U87MG), and hepatocellular carcinoma (Huh7). Arrow indicates bands of mature let-7a miRNA. 5S rRNA was used as loading control. **c** Firefly luciferase activities showed inverse correlation with expression level of endogenous let-7a which bind to the miRNA targeting sites in 3′UTR of firefly luciferase in each luc-let-7a-miRDREL. The firefly luciferase activities were normalized by those of *Renilla* luciferase. Data represent the mean values of three independent experiments ($n = 3$). Error bars in the graph represent ± standard deviation, and the P-values are based on a comparison of luc-let-7a-miRDREL to luc-Con-miRDREL. **d** and **f** Northern blot analysis of endogenous miR-7 **(d)** and miR-122 **(f)** miRNAs in various cell lines including human embryonic kidney (293T), non-small cell lung cancer (A549), metastatic lung cancer (NCI-H1299), pancreatic ductal adenocarcinoma (PANC-1), glioblastoma multiforme (U87MG), and hepatocellular carcinoma (Huh7). MiR-21 and let-7a probed nylon membranes in Fig. 1b and Supplementary Fig. 3b were stripped and reblotted with miR-7 and miR-

122-specific 5′-^{32}P-end-labeled DNA oligonucleotide probes, respectively. Arrow indicates band of mature miR-122. **e** and **g** Firefly luciferase activities represent expression level of endogenous miR-7 (A549 and Huh7) and miR-122 (293T and Huh7) which bind to the miRNA targeting sites in 3′UTR of firefly luciferase in each luc-miRDREL. The firefly luciferase activities were normalized by those of *Renilla* luciferase. Data represent the mean values of three independent experiments (n = 3). Error bars in the graph represent ± standard deviation, and the P-values are based on a comparison of luc-122-miRDREL to luc-Con-miRDREL. **P < 0.01, ***P < 0.001; NS, not significant.

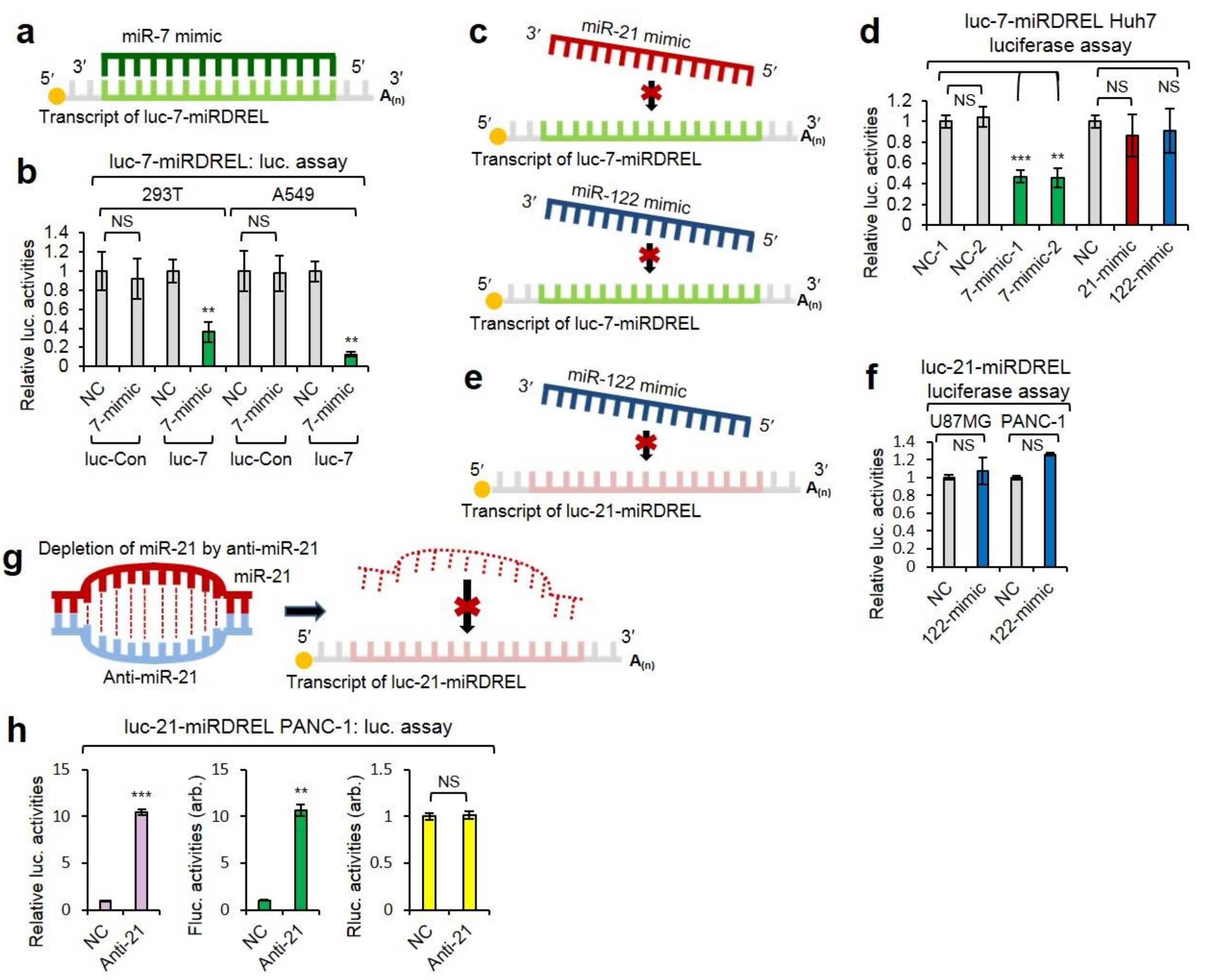

Supplementary Fig. 4 Analysis of miRDREL specificity with synthetic miRNA-mimics and antagomiRs. a Targeting principle of miR-7 mimic to the luc-7-miRDREL. **b** Inhibition of firefly luciferase activities indicate miR-7-mimic bind to the miR-7 targeting sites in luc-7-miRDREL. On the contrary to this, unaltered luciferase activities represent miR-mimic control (NC) does not bind to the luc-7-miRDREL which has miR-7 targeting sites. Data represent the mean values of three independent experiments (n = 3). Error bars in the graph represent ± standard deviation, and the P-values are based on a comparison of miR-7-mimics to the negative control (NC). **c-e** Nontargeting principle of miRNA mimics to the miRDRELs which bears irrelevant miRNA targeting sites. **d** Inhibition of firefly luciferase activities indicate each miR-7-mimic binds to the miRNA targeting sites in luc-7-miRDREL Huh7 cells. On the contrary to this, unaltered luciferase activities represent miR-mimics (21- and 122-mimics) do not bind to the luc-7-miRDREL which has noncompatible miRNA targeting sites. **f** luc-21-miRDREL U87MG and PANC-1 were transfected with synthetic 122-mimic or its relevant negative control oligomer (NC). Unaltered luciferase activities represent miR-mimic

control (NC) and miR-122-mimic do not bind to the luc-21-miRDREL which has miR-21 targeting sites. Data represent the mean values of three independent experiments ($n = 3$). Error bars in the graph represent ± standard deviation, and the P-values are based on a comparison of each mimic (7-mimic-1 and -2) to the negative control (NC-1). **g** Depletion strategy of the endogenous miR-21 with anti-miR-21 in cells. **h** Specific depletion of endogenous miR-21 via its synthetic anti-miR-21 transfection specifically induced derepression of firefly luciferase activities but not those of normalization control *Renilla* luciferase. Data represent the mean values of three independent experiments ($n = 3$). Error bars in the graph represent ± standard deviation, and the P-values are based on a comparison of anti-miR-21 to the negative control (NC). $^{**}P < 0.01$, $^{***}P < 0.001$; NS, not significant; arb., arbitrary unit.

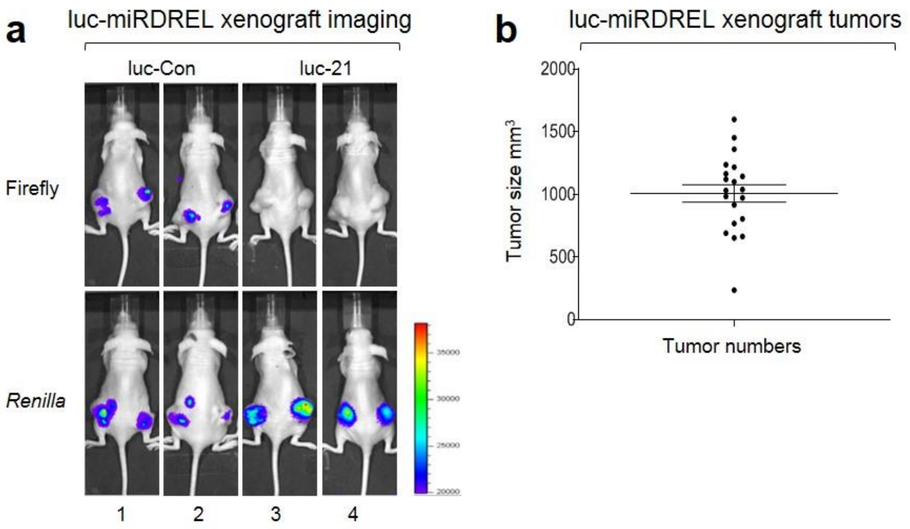

Supplementary Fig. 5 Luminescence imaging and distribution of size of luc-Con- and luc-21-miRDREL A549 mouse xenograft tumors. luc-Con- and luc-21-miRDREL A549 cells were subcutaneously injected into flank of nude mice. After four weeks, tumors of mice were assessed *in vivo* imaging with firefly and *Renilla* luciferase substrates. **a** luc-21-miRDREL showed no firefly luciferase activities which indicate translation repression of the firefly luciferase sensors by endogenous miR-21. **b** Dot graph represents distribution of formed tumor sizes after subcutaneous injection of luc-Con- and luc-21-miRDREL A549 cells. Tumors were sorted by size for normalization of experiments.

Supplementary References

1. Chapman, B.S., Thayer, R.M., Vincent, K.A. & Haigwood, N.L. Effect of intron A from human cytomegalovirus (Towne) immediate-early gene on heterologous expression in mammalian cells. *Nucleic Acids Res* **19**, 3979-3986 (1991).
2. Li, M., *et al.* Optimal promoter usage for lentiviral vector-mediated transduction of cultured central nervous system cells. *J Neurosci Methods* **189**, 56-64 (2010).
3. Ramezani, A., Hawley, T.S. & Hawley, R.G. Lentiviral vectors for enhanced gene expression in human hematopoietic cells. *Mol Ther* **2**, 458-469 (2000).
4. Salmon, P., *et al.* High-level transgene expression in human hematopoietic progenitors and differentiated blood lineages after transduction with improved lentiviral vectors. *Blood* **96**, 3392-3398 (2000).
5. Qin, J.Y., *et al.* Systematic comparison of constitutive promoters and the doxycycline-inducible promoter. *PLoS One* **5**, e10611 (2010).
6. Curtin, J.A., Dane, A.P., Swanson, A., Alexander, I.E. & Ginn, S.L. Bidirectional promoter interference between two widely used internal heterologous promoters in a late-generation lentiviral construct. *Gene Ther* **15**, 384-390 (2008).
7. Cormack, B.P., Valdivia, R.H. & Falkow, S. FACS-optimized mutants of the green fluorescent protein (GFP). *Gene* **173**, 33-38 (1996).
8. Shaner, N.C., *et al.* Improved monomeric red, orange and yellow fluorescent proteins derived from Discosoma sp. red fluorescent protein. *Nat Biotechnol* **22**, 1567-1572 (2004).
9. Lee, S.H., *et al.* The ubiquitin ligase human TRIM71 regulates let-7 microRNA biogenesis via modulation of Lin28B protein. *Biochim Biophys Acta Gene Regul Mech* **1839**, 374-386 (2014).
10. Kim, J.H. & Richter, J.D. Opposing polymerase-deadenylase activities regulate cytoplasmic polyadenylation. *Mol Cell* **24**, 173-183 (2006).
11. Yin, J., *et al.* DEAD-box RNA helicase DDX23 modulates glioma malignancy via elevating miR-21 biogenesis. *Brain* **138**, 2553-2570 (2015).
